# Extracellular Vesicle Packaged TDP43 Derived From ECs Exacerbates Cigarette Tar‐Related Atherosclerosis Progression via Enhancing Macrophage Extracellular Traps

**DOI:** 10.1002/advs.78001

**Published:** 2026-09-27

**Authors:** Xinxin Zhu, Yuwu Chen, Wei Meng, Biyi Xu, Junke Mou, Xiaolin Liu, Mengyang Wang, Qishuo Gu, Qianhui Sun, Man Li, Chen Zhao, Ming Zeng, Ying Lv, Boling Yi, Sheng Qu, Xiaoxuan Bai, Ji Li, Xing Luo, Sining Hu, Bo Yu, Haibo Jia

**Affiliations:** ^1^ Department of Cardiology the Second Affiliated Hospital of Harbin Medical University State Key Laboratory of Frigid Zone Cardiovascular Diseases (SKLFZCD) Harbin P. R. China; ^2^ The Key Laboratory of Myocardial Ischemia Chinese Ministry of Education Harbin P. R. China

**Keywords:** atherosclerosis, macrophage extracellular traps, MAMs, smoke, TDP43

## Abstract

Smoking acts as a critical risk factor for atherosclerosis (AS), but the precise molecular mechanisms remain unclear. We performed in vivo experiments with TAR‐injected ApoE^−/−^ mice and in vitro co‐culture of macrophages with extracellular vesicles (EVs) from TAR‐treated endothelial cells (ECs). Single‐cell RNA sequencing were used to explore the regulatory effects and molecular pathways of ECs‐derived EVs in smoke‐related AS. Cigarette TAR significantly stimulated ECs EV secretion and aggravated atherosclerotic plaques, while the EV inhibitor GW4869 alleviated these lesions. RNA‐seq indicated TAR facilitated AS by increasing macrophage extracellular traps (METs) formation. Combined in vivo and in vitro proteomics showed TDP43 was highly enriched in circulating EVs from smoking patients, TAR‐treated mice, and stimulated ECs. TDP43 knockdown alleviated TAR‐induced AS, accompanied by suppressed METs and inflammatory responses. Mechanistically, EV‐delivered TDP43 bound macrophage VDAC1 to induce mitochondria‐associated membranes (MAMs), triggering Ca^2+^ overload/METs; 2‐APB and BAPTA blocked this process. Retinoic acid (RA) might bind with TDP43 to inhibit MAMs, thereby reducing METs and mitigating smoke‐related AS. Cigarette TAR accelerates ASthrough EC EV‐packaged TDP43 partly, which binds VDAC1 to induce MAMs, calcium overload, METs, and inflammation. RA may serve as a putative protective molecule for the progression of smoking‐related atherosclerotic plaques.

## Graphical abstract

Cigarette tar triggers EC‐derived EVs carrying TDP43, which binds VDAC1 to boost MAMs, mitochondrial Ca^2+^ overload, and METs formation in macrophages, accelerating smoking‐related AS progression, while retinoic acid binds TDP43 to block this pathogenic cascade.

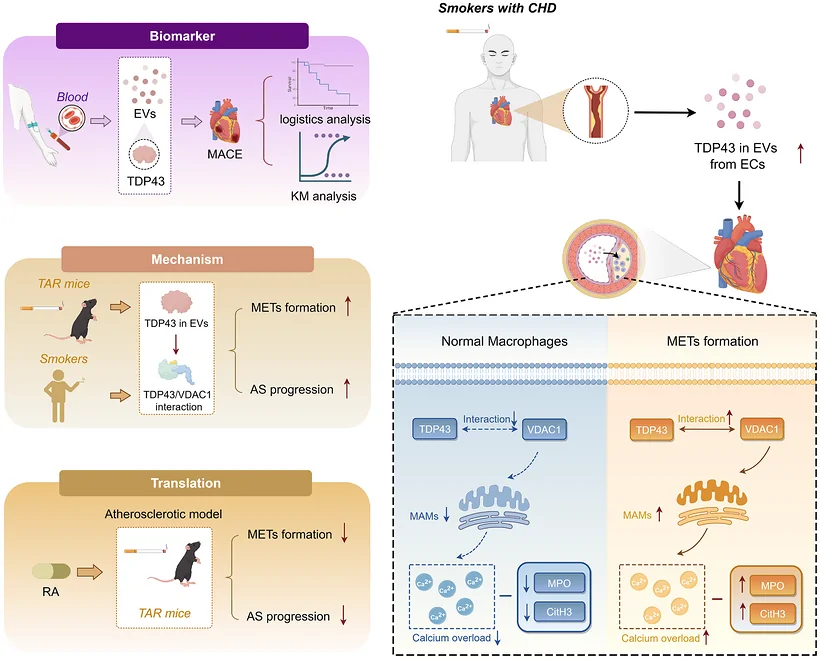

**Abbreviations**
ANOVA: One‐way analysis of variance
ApoE: Apolipoprotein E
AS: Atherosclerosis
BMI: Body mass index
CAD: Coronary artery disease
CETSA: Cellular thermal shift assay
Cf‐DNA: Circulating cell‐free DNA
CI: Confidence intervals
CitH: Citrullinated histones
CRP: C‐reactive protein
DARTS: Drug affinity responsive target stability
dsDNA: Double‐stranded DNA
ECG: Electrocardiogram
ECs: Endothelial cells
ETs: Extracellular traps
EVs: Extracellular vesicles
HD: High‐fat diet
HDL‐C: High‐density lipoprotein cholesterol
HRs: Hazard ratios
HUVECs: Human umbilical vein endothelial cells
IP3R: Inositol 1,4,5‐trisphosphate receptor
LDL‐C: Low‐density lipoprotein cholesterol
MACE: Major adverse cardiac events
MAMs: Mitochondria‐associated membranes
METs: Macrophage extracellular traps
MPO: Myeloperoxidase
NETs: Neutrophil extracellular traps
NTA: Nanoparticle tracking
PCI: Percutaneous coronary intervention
RA: Retinoic acid
RMSD: Root Mean Square Deviation
RMSF: Root Mean Square Fluctuation
RNA‐seq: RNA sequencing
ROS: Reactive oxygen species
SASA: Solvent Accessible Surface Area
SD: Standard deviation
STEMI: ST‐segment elevation myocardial infarction
TC: Total cholesterol
TDP43: Transactive response DNA binding protein of 43 kDa
TG: Triglycerides

## 1 Introduction

Atherosclerosis (AS) serves as the common pathological basis underlying numerous ischemic diseases, thereby posing a substantial threat to cardiovascular health [1, 2]. It has been well‐documented that smoking constitutes a primary risk factor contributing to the accelerated progression of AS [3, 4, 5, 6, 7, 8]. Also, empirical evidence has indicated that smoking enhances the instability of atherosclerotic plaques and elevates the incidence of adverse cardiovascular events [9, 10, 11]. Furthermore, the risk associated with previous smoking persists for up to two decades following cessation [12]. Nevertheless, the precise mechanisms through which smoking exacerbates the progression of AS remain largely elusive.

Extracellular traps (ETs) constitute a distinctive death pattern characterized by releasing double‐stranded DNA (dsDNA), myeloperoxidase (MPO), and citrullinated histones (CitH), which collectively form a reticular matrix implicated in inflammatory responses [13, 14, 15]. Several studies have demonstrated that various immune cells, including neutrophils, eosinophilics, and macrophages are capable of forming ETs [16, 17, 18, 19, 20]. A significant presence of ETs markers such as MPO and neutrophil elastase, have been detected in the atherosclerotic plaques of both humans and Apolipoprotein E knockout (ApoE^−^/^−^) mice [21, 22]. Further evidence demonstrated that the ETs contributed to the progression of AS via damaging endothelial cells (ECs), promoting the proliferation and migration of smooth muscle cells, activating platelets, and enhancing inflammatory responses [21, 23]. Current research predominantly focuses on the role of neutrophil extracellular traps (NETs) in the progression of AS and thrombosis [24, 25]. However, emerging data indicate that in various inflammatory diseases, macrophages also play a crucial role in modulating inflammatory responses and immune defense through the formation of macrophage extracellular traps (METs) [14, 26, 27]. Nonetheless, the role and regulatory mechanism of METs in the progression of AS remain to be fully elucidated.

Extracellular vehicles (EVs) are double‐membrane particles secreted by cells under both physiological and pathological conditions [28, 29], which are regarded as an important role in intercellular communication by encapsulating miRNAs, enzymes, and lipids [30, 31]. An increasing body of research has elucidated the involvement of EVs‐mediated intercellular communication in the progression of AS. Tian et al. have demonstrated that nicotine exacerbates the progression of AS plaques via enhancing the secretion of miR‐155 encapsulated within EVs derived from macrophages [32]. Similarly, Wang et al. also identified that miR‐21‐3p present in EVs from nicotine‐treated macrophages accelerates AS development by promoting vascular smooth muscle cell migration and proliferation through the targeting of PTEN [33]. Even so, the precise role and underlying mechanisms of EVs in the progression of AS plaques induced by smoking necessitate further investigation

Transactive response DNA binding protein of 43 kDa (TDP43) is ubiquitously expressed in neurons and glial cells, where it plays a pivotal role in neuroinflammatory diseases by influencing the stability of mRNA within the nucleus. It has been documented that TDP43 contributes to the neurodegenerative diseases progression by regulating mRNA alternative splicing and translocation, as well as influencing oxidative stress, and DNA damage [34]. Increasing evidence has shown that TDP43 present in the cytoplasm is also involved in immune regulation and the maintenance of oxidative homeostasis [35, 36]. Increasing evidence has indicated that TDP43 aggregates within the cytoplasm and facilitates neurodegeneration in amyotrophic lateral sclerosis [37]. Recent studies have reported that TDP43 aggregates in the cytoplasm, thereby facilitating neurodegeneration in amyotrophic lateral sclerosis [38]. Despite these findings, the precise effects and underlying mechanisms of TDP43 in AS remain inadequately understood.

In the current study, we investigated the role of EVs mediated communication between macrophages and endothelial cells in the progression of atherosclerotic plaques induced by smoking, Furthermore, we elucidated the potential mechanism by which cigarette TAR promotes atherosclerotic plaque progression through TDP43‐triggered METs.

## 2 Methods

### 2.1 Patients Study Design

(1) This study was approved by the Ethics Committee of the Second Affiliated Hospital of Harbin Medical University (Approval No. YJSDW2024‐047). All subjects provided informed consent prior to their inclusion in the study, and the experiments complied with the principles outlined in the Declaration of Helsinki. To compare the levels of TDP43 in circulating EVs between smoking and non‐smoking patients, we included 30 smokers and 30 non‐smokers diagnosed with coronary artery disease (CAD) at the Second Affiliated Hospital of Harbin Medical University, each with at least one coronary artery stenosis exceeding 50%. Participants were matched for age, sex, and risk factors including history of hypertension, diabetes, and hyperlipidemia. For each patient, 5 mL of peripheral blood was collected. The detailed description was provided in the Supporting Information. All these patients provided written informed consent. Inclusion criteria: (1) Diagnosis of stable or unstable coronary heart disease confirmed by coronary angiography, defined as at least one major epicardial coronary artery with luminal stenosis ≥50%. (2) Presence of either documented prior myocardial infarction (>3 months before enrollment), or typical angina with objective evidence of ischemia (e.g., positive stress test or fractional flow reserve ≤0.80). (3) 18–60 years. Exclusion criteria: (1) Acute or chronic inflammatory conditions (e.g., rheumatoid arthritis, systemic lupus erythematosus, inflammatory bowel disease, or active infection). (2) Pregnancy or lactation. (3) Known bleeding diathesis or concurrent anticoagulant therapy that contraindicates venipuncture (e.g., international normalized ratio >2.5). (4) Any condition that, in the investigator's opinion, would compromise patient safety or data integrity.

(2) 485 ST‐segment elevation myocardial infarction (STEMI) patients presented with acute myocardial infarction were recruited between January 2017 and September 2019 and were followed up prospectively for 5 years (Clinical Trials. gov ID: NCT03297164). STEMI was defined as continuous chest pain for > 30 min, ST‐segment elevation > 0.1 mV in at least two contiguous leads, or new left bundle‐branch block on the 12‐lead electrocardiogram (ECG), and elevated cardiac markers (troponin T/I or creatine kinase‐MB). Timely reperfusion was defined as first medical contact (FMC)‐to‐needle 30 min for patients undergoing thrombolytic therapy or FMC‐to‐wire crossing 120 min for patients undergoing primary percutaneous coronary intervention

(PCI). Family history of CHD was defined as an immediate relative receiving a diagnosis of having CHD before age 60 years. The criteria for selection were (1) patients diagnosed with STEMI, (2) time from the onset of chest pain symptoms to admission less than 6 h, (3) receiving percutaneous coronary revascularization intervention, and (4) signing an informed consent. Exclusion criteria for STEMI patients: (1) patients with malignancy; (2) autoimmune diseases such as systemic lupus erythematosus, rheumatoid arthritis, and compulsory spondylitis; (3) co‐infection with severe infection; (4) refusal to sign informed consent; and (5) hemodynamic instability during PCI. The detailed description was provided in the Supporting Information. The major adverse cardiac events (MACE) were a composite of all‐cause death, recurrent nonfatal MI, nonfatal stroke, or hospitalization due to HF.

### 2.2 Animal Studies

The study received approval from the Institutional Research Ethics Committee of Harbin Medical University (Approval No. YJSDW2024‐047). All animal experiments were designed, performed, and reported in accordance with the ARRIVE 2.0 guidelines. All animal experiments adhered to the guidelines of the National Institutes of Health for the care and use of laboratory animals. Male ApoE^−/−^ mice, aged eight weeks and on a C57BL/6 background, were procured from Beijing Vital River Laboratory Animal Technology Co., Ltd. (Beijing, China). These mice were provided with unrestricted access to food and water and were housed at the Animal Center of the Second Affiliated Hospital of Harbin Medical University under controlled conditions of 22°C ± 2°C temperature, 55% ± 5% relative humidity, and a 12‐h light/dark cycle. The body weight, blood glucose levels, and food intake of the mice were systematically monitored biweekly. The detailed description was provided in the Supporting Information.

### 2.3 Cell Experiments

The human monocytic THP‐1 cells (provided by the ATCC institution in the United States) were cultured in RPMI 1640 medium (provided by Gibco in the United States), with 10% fetal bovine serum (provided by Gibco in the United States) and 1% penicillin (provided by Beo‐Taim in China) added to the medium, and cultured in an environment at 37°C with 5% carbon dioxide saturation. HUVECs (purchased from the National Certified Cell Culture Repository in Shanghai, China) were cultured in the Dubecco modified Eagle's medium (DMEM; Gibco in New York State, Grand Isle Island) with 10% fetal bovine serum (FBS; Scientific Cell Company, San Diego) in an environment at 37°C with 5% carbon dioxide saturation. 100 µg/mL of cigarette TAR was used to stimulate human umbilical vein endothelial cells (HUVECs) for 24 h [39]. Additional methodological information is provided in the Supporting Information.

### 2.4 Single‐Cell Sequencing (scRNA‐seq) Analysis

For the scRNA‐seq, a total of 30 aortas were collected from 8‐week‐old male ApoE^−/−^ mice (15 ApoE^−/−^ mice in high‐fat diet (HD)+Tar group, 15 ApoE^−/−^ mice in the HD group). Based on a previous study [39], as for the HD+Tar group, 15 ApoE^−/−^ mice were subjected to 40 mg/kg/day cigarette TAR administration. Concurrently, these mice were maintained on HD (21% fat, 0.15% cholesterol; MD12015; Medicine Ltd., Jiangsu, China), for a duration of 16 weeks. Meanwhile, the remaining 15 ApoE^−/−^ mice (HD group) were also fed with HD for 16 weeks. Every 15 blood vessels were pooled to form a single sample for further scRNA‐seq analysis. Consequently, a total of two samples were utilized for scRNA‐seq, comprising one sample in the HD group and one sample in the HD+Tar group (1 vs. 1). The Seurat software (version 4.1.1) was employed for data integration and dimensional reduction. It is important to highlight that this scRNA‐seq profiling was specifically designed for exploratory visualization of the cellular composition of the aorta and to preliminarily assess the communication tendencies between ECs and macrophages. Given the pooled sample strategy without independent biological replicates, all differential signaling inferences derived from the scRNA‐seq data were subsequently validated through bulk transcriptomics and functional experiments, each conducted with multiple biological replicates. Detailed methodologies are provided in the Supporting Information.

### 2.5 Statistical Analysis

All quantitative data are presented as mean ± standard deviation (SD) from no fewer than three independent biological replicates. For normally distributed data between two groups, the two‐tailed Student's *t*‐test was applied. The Mann‑Whitney U test was adopted for datasets that did not conform to a normal distribution. One‐way analysis of variance (ANOVA) combined with the Bonferroni post‐hoc test was used for multiple intergroup comparisons. All statistical analyses were performed using SPSS 21.0 software, and a p‐value < 0.05 was defined as statistically significant. The detailed description was provided in the Supporting Information. The patients were divided into the high‐level TDP43 group and the low‐level TDP43 group based on the median level of TDP43 in their circulating EVs. For survival analysis, time‐to‐first event curves were generated by Kaplan‐Meier analysis, and compared using the log‐rank test. In addition, univariate and multivariable Cox proportional‐hazards regression models were used to estimate hazard ratios (HRs) and 95% confidence intervals (CI). Adjustments were made for baseline differences and potentially confounding prognostic factors. In adjusted model 2, age and sex were adjusted. In adjusted model 3, besides those factors, hypertension, diabetes, BMI and CRP were also adjusted. In adjusted model 4, besides those factors, the SYNTEX score was also adjusted.

## 3 Results

### 3.1 Cigarette TAR Exacerbated Atherosclerotic Lesion Progression in ApoE^−/−^ Mice Via Secreting EVs

**FIGURE 1:**
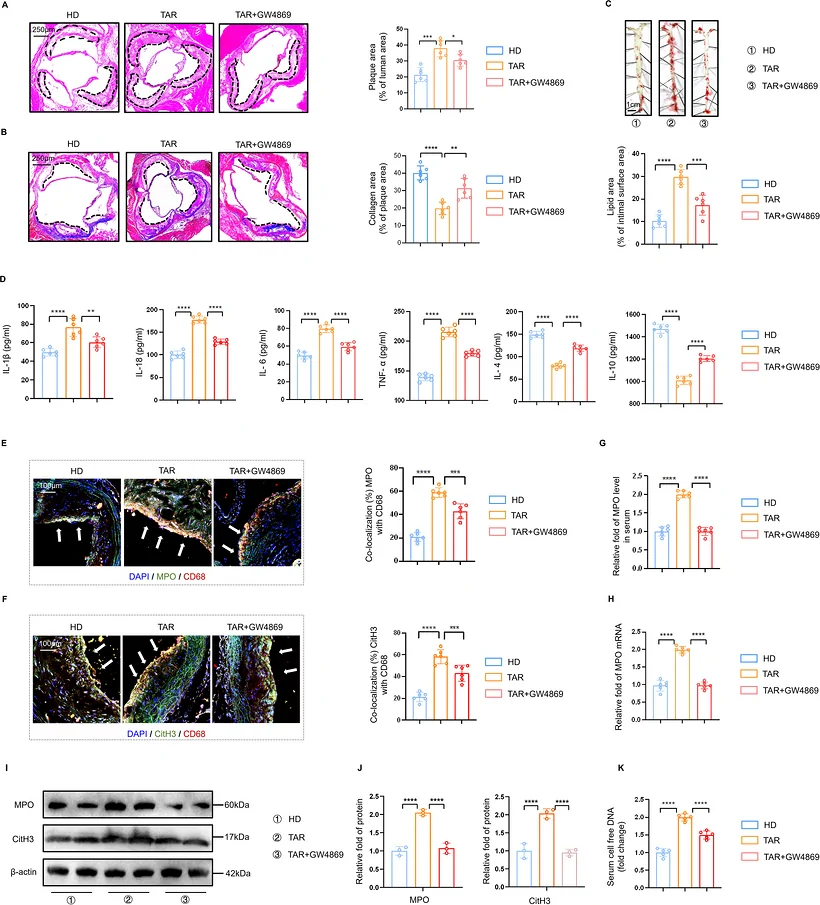
Cigarette TAR could exacerbate the progression of AS plaques in ApoE^−/−^ mice, while the EVs inhibitor GW4869 can reverse plaque progression in mice. (A) HE staining and quantitative analysis of ApoE^−/−^ mice (N = 6); (B) Masson staining and quantitative analysis of ApoE^−/−^ mice (N = 6); (C) Aortic Oil Red O staining and quantitative analysis of ApoE^−/−^ mice (N = 6); (D) Serum IL‐1β, IL‐18, IL‐6, TNF‐α, IL‐4 levels, IL‐10 levels (N = 6); (E‐F) Immunofluorescence results show co‐localization of MPO, CitH3 and CD68 (N = 6); (G) MPO serum level evaluation via ELISA (N = 6); (H) Total RNA was extracted from the cells to detect MPO mRNA levels (N = 6); (I, J) Western blot analysis and quantification of MPO and CitH3 protein levels (N = 3); (K) Levels of cf‐DNA in plasma of ApoE^−/−^ mice from the HD, TAR, and TAR+GW4869 groups (N = 5); Results are expressed as mean±SD. Comparisons of parameters were performed with one‐way ANOVA with a Bonferroni post hoc test. ^*^ *p* < 0.05, ^**^ *p* < 0.01, ^***^ *p* < 0.001, ^****^ *p* < 0.0001.

To elucidate the specific effects of EVs in smoke‐related atherosclerotic plaque progression, ApoE^−/−^ mice were allocated into three distinct groups: the HD group, TAR group, and TAR+GW4869 (inhibitor of EVs secretion) group, The results of H&E and Oil Red O staining demonstrated a significant increase in plaque area at the aortic root and lipid accumulation within the arteries in the TAR group compared with the HD group. This increase was significantly mitigated by the administration of GW4869 (Figure 1A,C). Furthermore, Masson's trichrome staining indicated a reduction in collagen content within atherosclerotic plaques in the TAR group relative to the HD group, a decrease that was significantly alleviated by GW4869 treatment (Figure 1B). In addition, serum levels of inflammatory cytokines were evaluated. The concentrations of pro‐inflammatory cytokines, including IL‐1β, IL‐18, IL‐6, and TNF‐α, were found to be elevated in the TAR group. Conversely, these pro‐inflammatory factors exhibited a significant reduction in the GW4869 group. The serum concentrations of anti‐inflammatory cytokines IL‐4 and IL‐10 were also measured, indicating that TAR administration could lead to decreased serum levels of IL‐4 and IL‐10 in ApoE^−/−^ mice. Nevertheless, following GW4869 administration, there was a significant increase in the serum levels of these cytokines (Figure 1D). Furthermore, exposure to cigarette TAR was associated with elevated serum levels of triglycerides (TG), total cholesterol (TC), and low‐density lipoprotein cholesterol (LDL‐C) (Figure S1A–C), along with a decreased level of high‐density lipoprotein cholesterol (HDL‐C) (Figure S1D). Notably, GW4869 treatment did not ameliorate the lipid metabolism disorder. Hence, the above findings indicated that TAR could aggravate immune inflammatory responses via promoting the release of EVs, thereby facilitating the progression of AS.

### 3.2 Cigarette TAR Promoted METs Formation via Enhancing the Secretion of EVs in ECs

ECs dysfunction is recognized as the initial pathological process in AS and constitutes a major source of EVs throughout the progression of the disease [32]. Consequently, the EVs isolated from the supernatant of ECs exposed to TAR were subjected to analysis. Electron microscopy was utilized to assess the morphology and diameter of the EVs, thereby confirming their successful isolation (Figure S2A). Western blot analysis further demonstrated a marked upregulation in the expression of EV markers (*Alix, TSG101*, and *CD63*) in the TAR‐treated group (Figure S2B). Furthermore, nanoparticle tracking (NTA) analysis provided a vesicle size distribution profile, revealing that TAR administration promoted increased EV secretion (Figure S2C), indicating that TAR could mediate EVs secretion from ECs. To elucidate the potential mechanisms through which EVs released by ECs under TAR stimulation influence AS, RNA sequencing (RNA‐seq) was utilized to analyze the aortic tissue of mice exposed to TAR. The RNA‐seq results indicated a significant upregulation of MPO, a critical gene associated with the formation of extracellular traps. Furthermore, Gene Ontology analysis (Up‐regulated genes) demonstrated a notable enrichment of functions related to macrophage activation and DNA secretion in the TAR group compared with the HD group (Figure S3A–D). Also, the EVs released by ECs following TAR treatment could be phagocytosed by macrophages (Figure S4). Based on these findings, we hypothesized that TAR might facilitate the release of EVs from ECs, thereby promoting the formation of METs.

Subsequently, we assessed the expression levels of macrophage METs‐related markers, specifically MPO and CitH3, within the atherosclerotic plaques of ApoE^−/−^mice treated with TAR. Our findings indicated that the expression of MPO and CitH3 in the aortic root atherosclerotic plaques of TAR‐treated mice was significantly elevated compared with the HD group. Furthermore, treatment with GW4869 markedly inhibited the expression of MPO and CitH3 of macrophages within atherosclerotic plaques (Figure 1E,F). These observations were corroborated by PCR/Western Blot/ELISA analyses, utilizing magnetic bead sorting of aortic macrophages in ApoE^−/−^ mice (Figure 1G–J). Furthermore, the results of immunofluorescence/HE staining in coronary artery disease (CHD) patients with or without smoking history suggested that the smoking group presented a larger plaque lesion area and increased METs formation within carotid atherosclerotic plaque (Figure S3E,F). Additionally, we evaluated circulating cell‐free DNA (cf‐DNA) in the plasma of ApoE^−/−^ mice across the HD, TAR, and TAR+GW4869 groups. The analysis revealed that cf‐DNA levels in the TAR group were significantly higher than those in the HD group, with GW4869 effectively attenuating this increase (Figure 1K).

**FIGURE 2:**
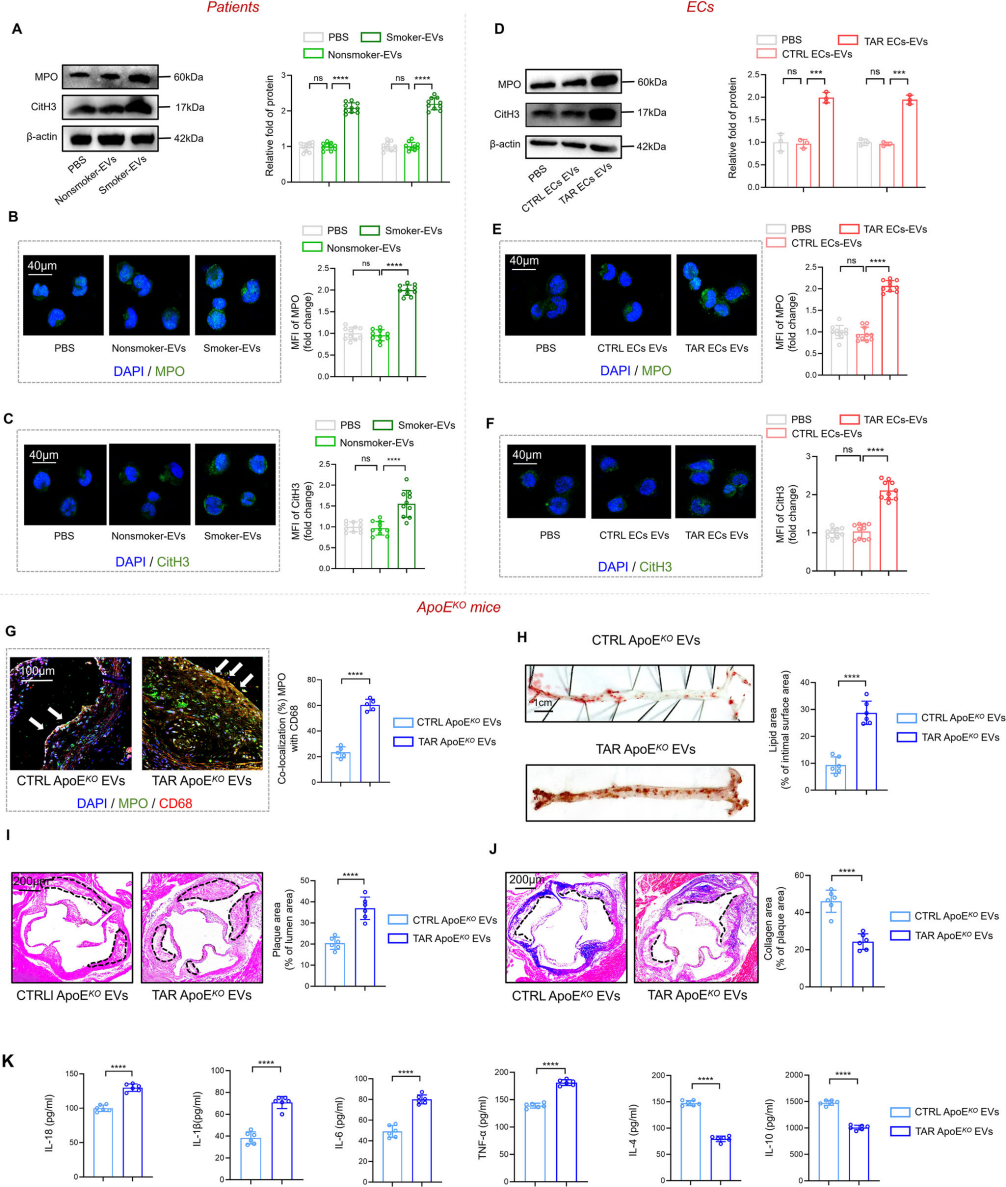
TAR promote METs formation via enhancing the secretion of EVs in ECs, ApoE^−/−^mice and patients. (A) Detection of protein expression levels of MPO and CitH3 in macrophages from smokers and nonsmokers by Western Blot (N = 10); (B, C) Immunofluorescence co‐staining of MPO and CitH3 with DAPI in plaques from smokers and nonsmokers (N = 10); (D) Western blot analysis was used to detect the protein expression levels of MPO and CitH3 in EVs derived from endothelial cells in the control group and the TAR‐treated group (N = 3); (E, F) Immunofluorescence co‐staining of MPO and CitH3 with DAPI in control and cigarette TAR‐stimulated endothelial cells; (G) Immunofluorescence co‐staining of MPO with DAPI in control and cigarette ApoE^−/−^ mice (N = 5); (H) Aortic Oil Red O staining and quantitative analysis of ApoE‐/‐mice (N=6); (I) HE staining and quantitative analysis of ApoE^−/−^mice (N = 6); (J) Masson staining and quantitative analysis of ApoE^−/−^mice (N = 6); (K) Serum IL‐1β, IL‐18, IL‐6, TNF‐α, IL‐4 levels, and IL‐10 levels (N = 6); Results are expressed as mean±SD. Comparisons of parameters were performed with one‐way ANOVA with Bonferroni post hoc test and Student's test. ^***^ *p* < 0.001, ^****^ *p* < 0.0001.

Furthermore, to investigate whether ECs‐derived EVs treated with TAR contributed to the enhanced formation of METs, EVs were isolated from the plasma of smokers and non‐smokers with CHD, and equivalent quantities were used to incubate macrophages. Western blot and immunofluorescence analyses indicated a significant upregulation of MPO and CitH3 expression in EVs derived from the serum of smokers compared with non‐smokers (Figure 2A–C). These findings were substantiated by in vitro co‐culture experiments (EV extraction from ECs with/without TAR treatment) and in vivo studies (EV extraction and packaging in ApoE^−/−^ mice with/without TAR treatment) (Figure 2D–G). Additionally, circulating EVs from ApoE^−/−^ mice with TAR treatment were observed to significantly exacerbate the progression of AS, including increased plaque area/lipid deposition (Figure 2H), decreased collagen content and an aggravated inflammatory response (Figure 2I–K), while circulating EVs from ApoE^−/−^ mice with TAR treatment did not result in lipid metabolism disorder (Figure S5). Moreover, the results of exploratory 10× single‐cell transcriptome sequencing (ApoE^−/−^ mice aorta with/without TAR treatment) indicated that there was an upward trend in ECs‐macrophage interaction probability upon TAR treatment (Figures S6 and S7). Collectively, these results suggested that TAR could facilitate the development of AS through METs formation mediated by EC‐derived EVs.

### 3.3 TDP43 Mediated METs Formation Induced by TAR Treated‐ECs Derived EVs

To elucidate the potential mechanism through which ECs‐derived EVs mediate METs formation under TAR exposure, we conducted a comparative proteomic profiling of EVs proteins isolated from three biologically relevant sources: Plasma from CHD patients with or without a smoking history; Plasma from TAR‐exposed vs. control ApoE^−/−^ mice; EVs from ECs treated with or without TAR. Integrated analysis identified TDP43 as a consistently upregulated EVs protein across all three cohorts (Figure S8). This elevated expression in EVs was subsequently validated at both the protein level and mRNA level (Figure S9), suggesting that TDP43 might serve as the crucial role in the formation of METs induced by TAR treated‐ECs derived EVs.

**FIGURE 3:**
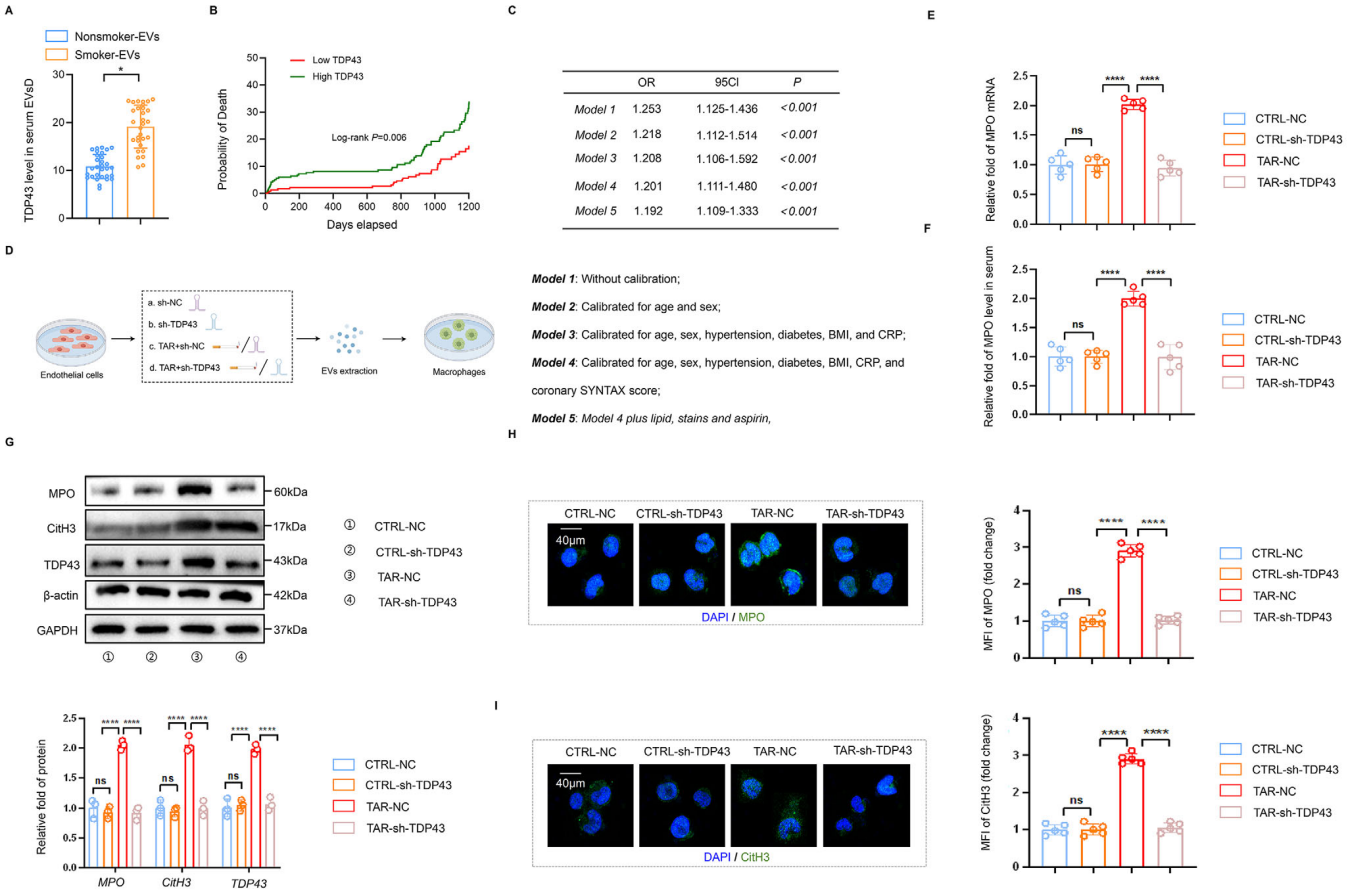
TDP43 mediates METs formation induced by TAR treated‐ECs derived EVs. (A) Levels of TDP43 in plasma EVs of smoking and non‐smoking patients (N = 30); (B) Kaplan‐Meier curve analysis of survival rates between low and high TDP43 level groups; (C) Logistic regression analysis of TDP43 in plasma EVs and MACE; (D) EVs derived from cigarette TAR‐stimulated ECs were isolated and incubated with macrophages; (E) Total RNA was extracted from the cells to detect MPO mRNA levels (N = 5); (F) MPO serum level evaluation via ELISA (N = 5); (G) Western blot analysis and quantification of the protein expression levels of MPO, CitH3, and TDP43 (N = 3); (H‐I) Co‐immunofluorescence staining of MPO/CitH3 and DAPI (N = 6); Results are expressed as mean±SD. Comparisons of parameters were performed with one‐way ANOVA with Bonferroni post hoc test and Student's test. ^*^ *p* < 0.05, ^****^ *p* < 0.0001.

Subsequently, to evaluate the intracellular localization of TDP43, we conducted a protease protection assay of extracellular vesicles, indicating that TDP43 was mainly located inside the EVs (Figure S10). Also, to further investigate the clinical relationship between TDP43 and smoking, we collected plasma samples from patients diagnosed with ST‐segment elevation myocardial infarction, both with and without a history of smoking, and subsequently extracted EVs. The results of the ELISA analysis indicated that the concentration of TDP43 in plasma EVs was significantly elevated in patients with a history of smoking compared with the non‐smoking patients (Figure 3A). Subsequently, we categorized the patients into high TDP43 and low TDP43 groups based on the median TDP43 levels in their EVs. Kaplan‐Meier curve analysis demonstrated that the survival rate of patients in the High TDP43 group was notably lower than that of the low TDP43 group (Figure 3B). Additionally, we obtained plasma EVs were isolated from patients with ST‐elevation myocardial infarction (STEMI) and a 5‐year follow‐up to monitor major adverse cardiovascular events (MACE) was conducted. The results showed that the increased TDP43 level in circulating EVs was associated with the incidence of MACE events (OR = 1.253 per 5 ng/µg, P<0.05). This association persisted even after adjusting for confounding variables such as age, gender, hypertension, diabetes, body mass index (BMI), C‐reactive protein (CRP), and coronary SYNTAX score (Figure 3C). These results provide preliminary, hypothesis‐generating evidence that the level of TDP43 in circulating EVs might be associated with the occurrence of MACE in patients with myocardial infarction, although further validation is warranted.

Also, to further elucidate the specific role of TDP43 in the formation of METs, ECs transfected with sh‐NC or sh‐TDP43 were exposed to cigarette TAR. EVs were subsequently collected and introduced into macrophages for a 48‐h co‐culture period (Figure 3D). Total RNA was extracted from the macrophages to assess MPO mRNA levels, revealing a significant reduction in MPO mRNA levels in the TAR‐sh‐TDP43 group compared with the TAR‐sh‐NC group (Figure 3E). These results were corroborated through western blot, confocal immunofluorescence, and ELISA analyses (Figure 3F–I and Figure S11). While after exogenous administration of TDP43 overexpression, the above indexes were significantly restored (Figure S12). Consequently, we speculate that TDP43 might be one of the contributing factors in the macrophage extracellular trap network triggered by Tar‑treated ECs‑derived EVs.

### 3.4 TDP43 Knockdown in ECs/ECs‐Derived EVs Alleviated the Effect on the Progression of TAR‐Related Atherosclerotic Plaque

**FIGURE 4:**
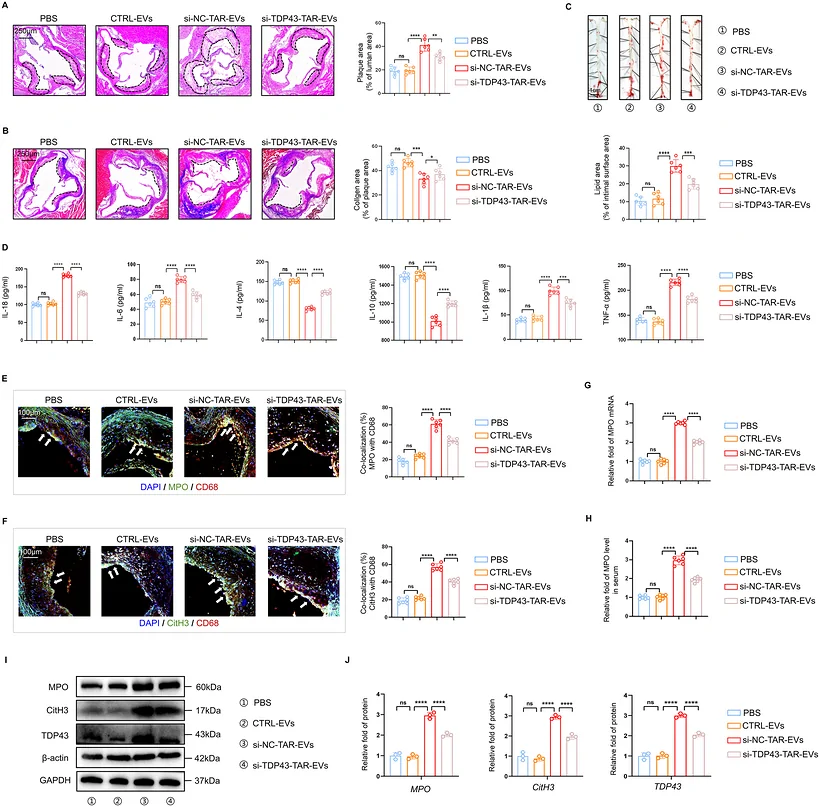
TDP43 knockdown in ECs/ECs‐derived EVs alleviates the effect on the progression of TAR‐related atherosclerotic plaque. (A) HE staining and quantitative analysis of ApoE^−/−^ mice (N = 6); (B) Masson staining and quantitative analysis of ApoE^−/−^ mice (N = 6); (C) Aortic Oil Red O staining and quantitative analysis of ApoE^−/−^ mice (N = 6); (D) Serum IL‐1β, IL‐18, IL‐6, TNF‐α, IL‐4, and IL‐10 levels (N = 6); (E, F) Immunofluorescence shows co‐localization of MPO, CitH3 and CD68 (N = 6); (G) Total RNA was extracted from cells to detect MPO mRNA levels (N = 6); (H) MPO serum level evaluation via ELISA (N = 6); (I, J) Western blot analysis and quantification of protein expression levels of MPO, CitH3, and TDP43 (N = 3); Results are expressed as mean±SD. Comparisons of parameters were performed with one‐way ANOVA with Bonferroni post hoc test. ^*^ *p* < 0.05, ^**^ *p* < 0.01, ^***^ *p* < 0.001, ^****^ *p* < 0.0001.

To further elucidate the role of TDP43 contained in EVs in the progression of atherosclerotic plaques, EVs derived from TDP43 knockdown ECs were isolated and administered to ApoE^−/−^ mice via tail vein injection. Subsequent analysis of arterial tissues from these mice demonstrated that the si‐TDP43‐TAR‐EVs group exhibited a significant reduction in plaque area at the aortic root compared with that in the si‐NC‐TAR‐EVs group (Figure 4A,C). Additionally, Masson staining indicated a significant increase in collagen content within the atherosclerotic plaques of the si‐TDP43‐TAR‐EVs group relative to the si‐NC‐TAR‐EVs group (Figure 4B). Furthermore, TDP43 knockdown was associated with a mitigation of the inflammatory response in ApoE^−/−^ mice, as evidenced by decreased levels of pro‐inflammatory cytokines (IL‐1β, IL‐18, IL‐6, and TNF‐α) and increased levels of anti‐inflammatory cytokines (IL‐4, IL‐10) (Figure 4D), while TDP43 knockdown did not improve the lipid metabolism disorder (Figure S13). Western blotting, PCR, ELISA and immunofluorescence staining results suggested that the si‐TDP43‐TAR‐EVs group exhibited reduced formation of METs compared with the si‐NC‐TAR‐EVs group (Figure 4E–J). Furthermore, the assessment of cf‐DNA in the plasma of ApoE^−/−^ mice revealed that the expression levels of cf‐DNA in the si‐NC‐TAR‐EVs group were significantly higher compared with that in the si‐TDP43‐TAR‐EVs group (Figure S14). Similar results were observed in ApoE^−/−^ mice subjected to HD+TAR administration and treated with AAV‐Tie2‐sh‐TDP43/AAV‐Tie2‐sh‐NC (Figures S15–S17), while ECs‐specific TDP43 knockdown did not improve the lipid metabolism disorder (Figure S18). These findings indicated that the knockdown of TDP43 in ECs‐derived EVs or ECs could significantly inhibit the formation of METs and mitigate the progression of atherosclerotic plaques induced by TAR administration.

### 3.5 TDP43 Derived From TAR‑Treated EC‐Derived EVs Regulates METs Formation via the VDAC1‑MAMs‑Mitochondrial Calcium Overload Axis

**FIGURE 5:**
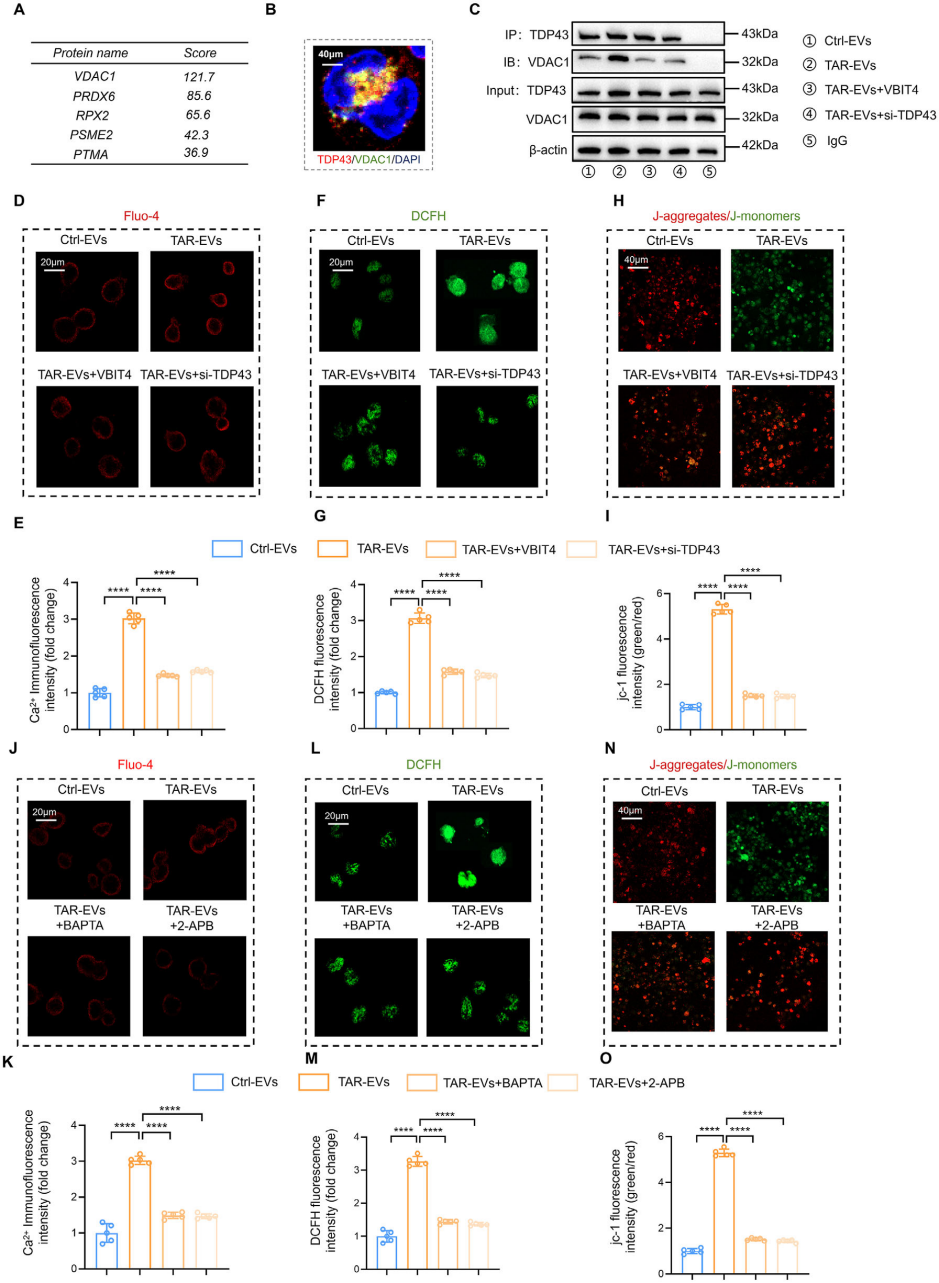
TDP43 derived from TAR‑treated EC‐derived EVs regulates METs formation via the VDAC1‑MAMs‑mitochondrial calcium overload axis. (A) IP/MS is used to study the binding proteins of TDP43; (B) Immunofluorescence co‐localization staining of TDP43 and VDAC1; (C) Co‐immunoprecipitation was used to assess the binding of TDP43 to VDAC1 across the four groups (N = 3); (D, E) Fluo‐4 fluorescent probe was used to detect intracellular and mitochondrial Ca^2^^+^ levels (N = 5); (F, G) DCFH fluorescent probe was used to detect intracellular ROS levels (N = 5); (H, I) JC‐1 fluorescent probe was used to detect mitochondrial membrane potential changes (N = 5); (J, K) Fluo‐4 fluorescent probe was used to detect intracellular and mitochondrial Ca^2^^+^ levels(N = 5); (L, M) DCFH fluorescent probe was used to detect intracellular ROS levels (N = 5); (N, O) JC‐1 fluorescent probe was used to detect mitochondrial membrane potential changes (N = 5); Results are expressed as mean±SD. Comparisons of parameters were performed with one‐way ANOVA with Bonferroni post hoc test and Student's test. ^****^ *p* < 0.0001.

To further investigate the mechanism by which TDP43 in TAR‐treated EC‐derived EVs mediated the formation of METs, mass spectrometry analysis was conducted to identify proteins bound to TDP43, the results of which suggested that the binding score of VDAC1 was the highest (Figure 5A). Also, the interaction between VDAC1 and TDP43 was further validated by confocal immunofluorescence and Co‐IP experiments (Figure 5B and Figure S19A). As the voltage‐dependent anion channel protein located in the outer mitochondrial membrane, VDAC1 was regarded as the crucial role in regulating intracellular calcium homeostasis and mitochondrial function [40]. In addition, mitochondria‐associated endoplasmic reticulum membranes (MAMs) are intracellular subcellular structures where mitochondria and endoplasmic reticulum are interconnected, and MAMs have been shown to have a role in regulating extracellular trapping networks. Previous studies have found that VDAC1 regulates MAMs formation by interacting with IP3R and GRP75 [41, 42]. Hence, we hypothesized that TDP43 in TAR‐treated ECs‐derived EVs increased METs formation by binding to VDAC1 to increase MAMs formation.

Immunofluorescence analysis demonstrated that treatment with TAR on ECs‐derived EVs markedly enhanced the presence of MAMs in macrophages. Conversely, the knockdown of TDP‐43 of ECs or administration of VBIT‐4, a specific inhibitor of VDAC1, significantly reduced MAMs, as illustrated in Figure S19F. Co‐IP assays further revealed that VBIT‐4 inhibited the interaction between TDP43 and VDAC1 (Figure 5C). In view of previous literature, MAMs facilitated the transfer of calcium ions from the endoplasmic reticulum to mitochondria via the inositol 1,4,5‐trisphosphate receptor (IP3R), leading to calcium overload, a critical factor in the induction of mitochondrial extracellular traps (METs) [36, 37]. Consequently, we investigated intra‐mitochondrial calcium ion and reactive oxygen species (ROS) levels, observing that TAR‐EVs significantly elevated both parameters. However, this effect was mitigated by either TDP‐43 knockdown or VDAC1 inhibition (Figure 5D–G and Figure S20A–D). Additionally, we assessed mitochondrial membrane potential and METs formation markers. Our findings indicated that either TDP‐43 knockdown or VDAC1 inhibition significantly ameliorated the reduction in mitochondrial membrane potential and METs formation (Figure 5H,I and Figure S19B,C,F,G). Comparable outcomes were also observed following treatment with 2‐APB, an IP3R inhibitor, and BAPTA, a calcium ion chelator (Figure 5J–O and Figures S19D,E,H and S20E–H). In addition, we also performed molecular docking analysis of TDP43 and VDAC1 and found that they bound at the aa145‐aa211 region. Subsequently, we performed a truncating experiment in which VDAC1 145–211 was knocked out and transfected into macrophages with wild‐type VDAC1. Results showed that deletion of VDAC1 at 145–211 failed to bind to TDP43 and mediate MAM formation (Figure S21). Hence, the results mentioned above indicated that TDP43 in EVs from TAR‑treated ECs modulated METs formation through the VDAC1‑MAMs‑mitochondrial calcium overload pathway.

### 3.6 RA Could Possibly Bind With TDP43 via Virtual Drug Screening

**FIGURE 6:**
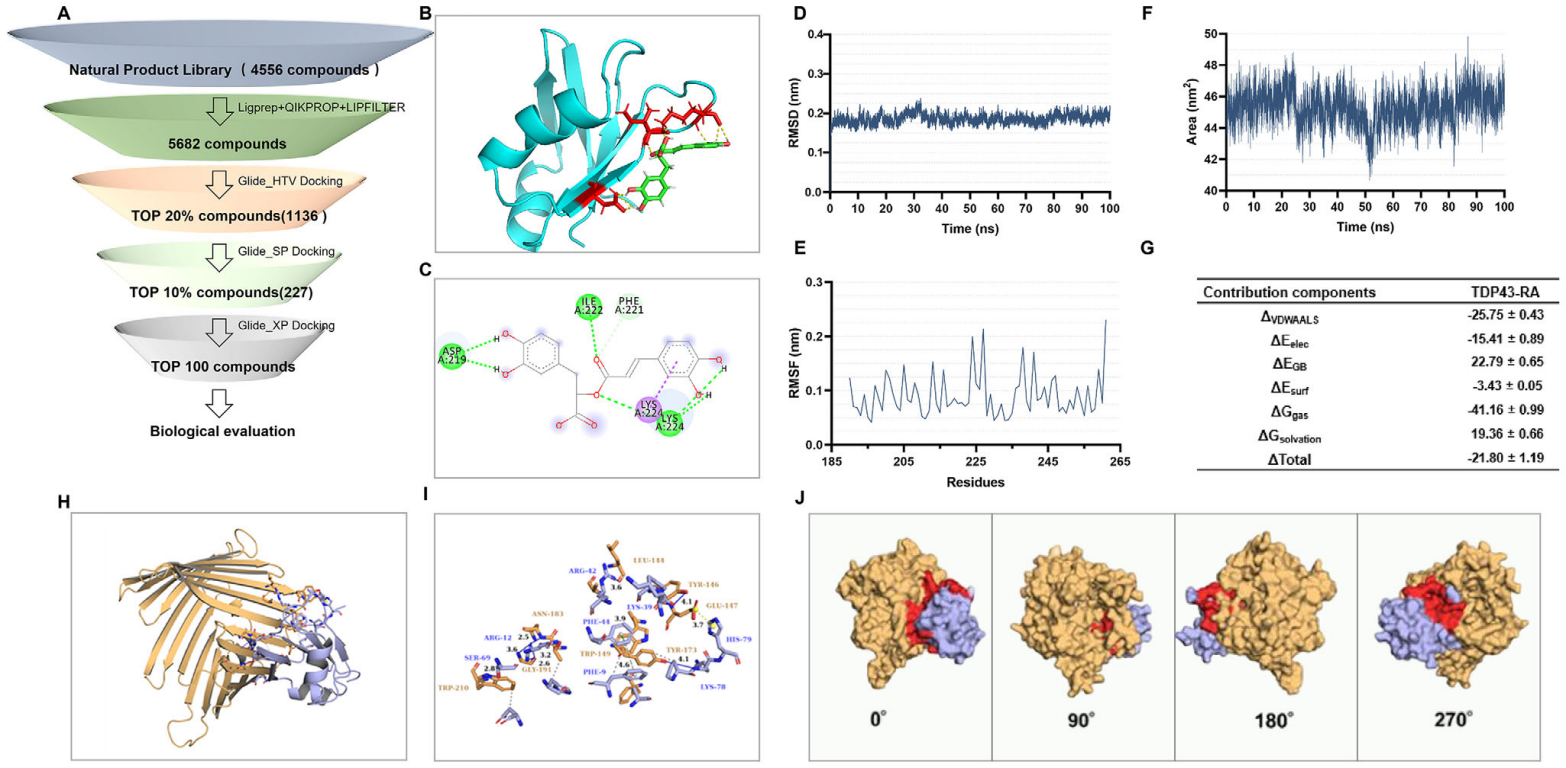
Virtual drug screening reveals RA binds to TDP43 and molecular docking of VDAC1 with TDP43. (A) Schematic diagram of virtual screening for small molecules binding to TDP43; (B, C) Molecular docking of TDP43 with RA; (D, E) RMSD and RMSF analysis reveal the binding stability of TDP43 and RA; (F) SASA analysis reveals the contact interface between RA and TDP43; (G) Composition of the binding force between RA and TDP43; (H) Molecular docking diagram of VDAC1 and TDP43; (I) Specific amino acid sites of protein binding; (J) Binding regions of VDAC1 and TDP43 from different perspectives.

In pursuit of identifying potential therapeutic agents targeting TDP43, we undertook a screening of small molecule compounds aimed at TDP43, guided by both the screening outcomes and the compounds’ biological characteristics. Retinoic Acid (RA) emerged as a candidate for further investigation (Figure 6A). Molecular docking analyses revealed that RA could interact with TDP43 via in silico‐predicted interaction, exhibiting a binding energy of ‐85.65 kcal/mol (Figure 6B,C). Subsequently, we employed molecular dynamics simulations to assess the stability of the RA‐TDP43 interaction via computational prediction. The Root Mean Square Deviation (RMSD) analysis demonstrated that the RA‐TDP43 complex attained a stable conformation after approximately 5 nanoseconds (Figure 6D). Notably, the Root Mean Square Fluctuation (RMSF) values for residues interacting with RA, particularly near positions 205, 225, and 245, remained low, signifying a robust binding interaction (Figure 6E). Concurrently, the average Solvent Accessible Surface Area (SASA) was determined to be 45.30±1.06 nm^2^, with minimal fluctuations, suggesting that the exposed protein surface region maintained stability throughout the simulation, without significant alterations in hydrophobic or hydrophilic properties due to the small molecule binding, further underscoring the adaptability and stability of the complex system within an aqueous solution environment (Figure 6F). These results collectively suggested that RA might have the potential to bind TDP43.

To quantify the binding affinity, we computed the binding free energy. The findings revealed that the total binding free energy, ΔTotal, was −21.80 ± 1.19 kcal/mol, a notably negative value that signifies a strong attractive interaction between TDP43 and RA, indicative of high affinity. Specifically, the van der Waals forces contributed ΔVDWAALS of −25.75 ± 0.43 kcal/mol, while the electrostatic forces contributed ΔEelec of −15.41 ± 0.89 kcal/mol, which further enhanced the binding strength through effective charge complementarity. The solvation free energy, ΔGsolvation, was 19.36 ± 0.66 kcal/mol; although positive, it was counterbalanced by the gas‐phase free energy, ΔGgas, of −41.16 ± 0.99 kcal/mol. Within the solvation energy, the polar solvation energy, ΔEGB, was 22.79 ± 0.65 kcal/mol, and the surface tension contribution, ΔEsurf, was −3.43 ± 0.05 kcal/mol. The analysis of these contributions demonstrated that the formation of the complex was energetically favorable, primarily driven by van der Waals and electrostatic interactions, while the solvation effect exerted a negative influence but did not significantly diminish the overall binding affinity. These findings were corroborated by the RMSD and hydrogen bond analyses, collectively affirming the potential of RA as an inhibitor (Figure 6G). Furthermore, molecular docking studies conducted on TDP43 and VDAC1 revealed multiple binding sites for the TDP43/VDAC1 complex. These binding regions were observable from various angles (0, 90, 180, and 270 degrees, etc.) (Figure 6H–J).

Furthermore, the cellular thermal shift assay and drug affinity responsive target stability were employed to corroborate this direct interaction (Figure S22A–C). Furthermore, micro‐scale thermophoresis analysis unequivocally demonstrated a direct interaction between RA and TDP43, characterized by a strong binding affinity (Kd = 45 µm) (Figure S22D).

### 3.7 RA Inhibited METs Formation and Alleviated Atherosclerotic Lesions via TAR‑EVs‑Mediated Mitochondrial‑ER Coupling and Calcium Overload

**FIGURE 7:**
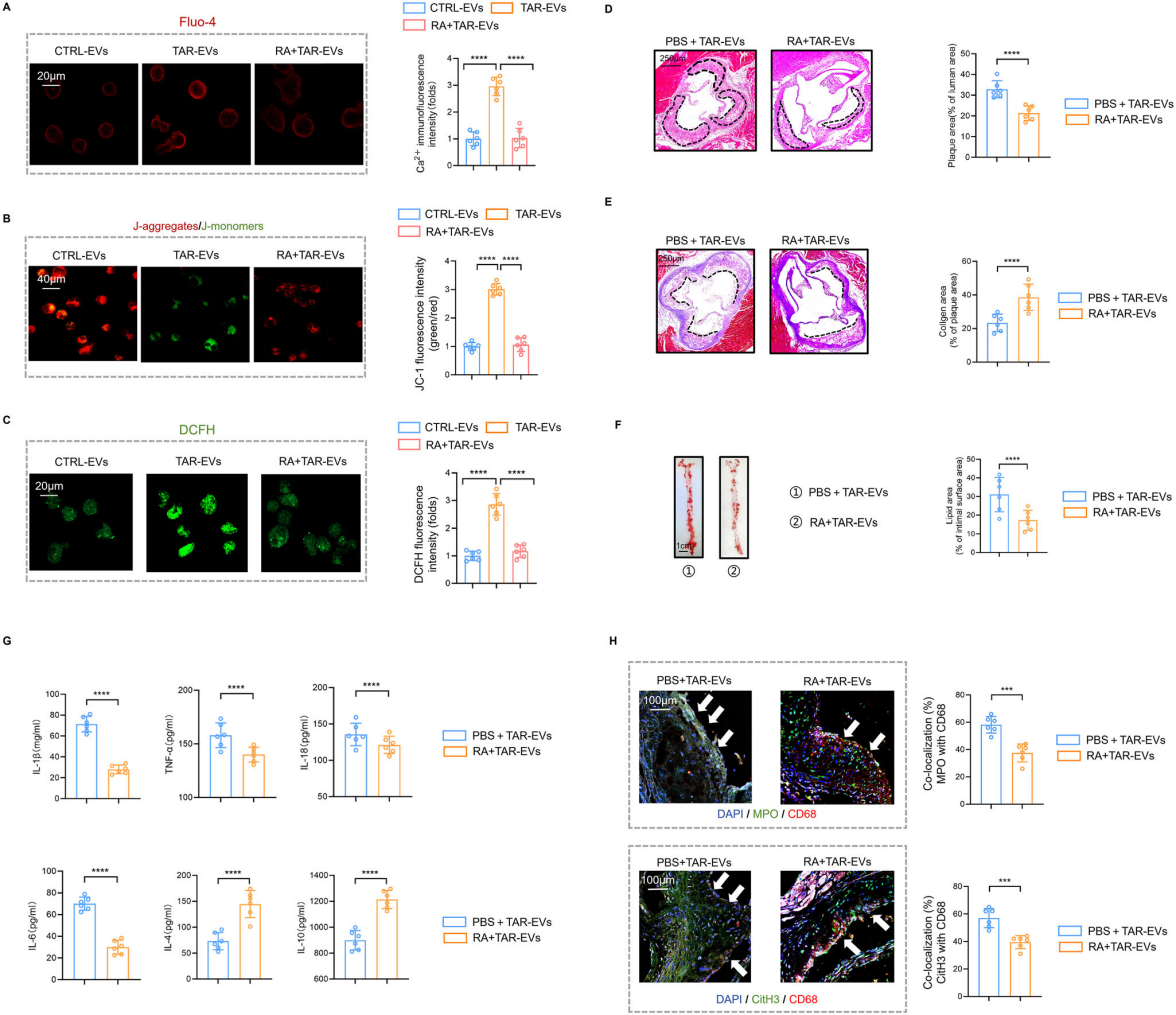
RA inhibits METs formation and alleviates atherosclerotic lesions via TAR‑EVs‑mediated mitochondrial‑ER coupling and calcium overload. (A) Fluo‐4 fluorescent probe was used to detect intracellular and mitochondrial Ca^2^^+^ levels (N = 6); (B) DCFH fluorescent probe was used to detect intracellular ROS levels (N = 6); (C) JC‐1 fluorescent probe was used to detect mitochondrial membrane potential changes (N = 5); (D) HE staining and quantitative analysis of ApoE^−/−^ mice (N = 6); (E) Masson staining and quantitative analysis of ApoE^−/−^ mice (N = 6); (F) Aortic Oil Red O staining and quantitative analysis of ApoE^−/−^ mice (N = 6); (G) Serum IL‐1β, IL‐18, IL‐6, TNF‐α, IL‐4 levels, IL‐10 levels (N = 6); (H) Immunofluorescence results show co‐localization of MPO, CitH3 and CD68 (N = 6); Results are expressed as mean±SD. Comparisons of parameters were performed with one‐way ANOVA with Bonferroni post hoc test and Student's test. ^***^ *p*<0.001, ^****^ *p* < 0.0001.

To investigate the specific role of RA in the progression of smoke‐related AS, we collected the supernatant EVs from ECs (PBS/TAR treatment) and co‐cultured them with macrophages, which were administered with RA. The results showed that the expression of key proteins (MPO/CitH3) in METs, MAM formation, the calcium ion concentration, and the level of reactive oxygen species of the RA‐TAR‐EVs treatment group were significantly lower than those of the TAR‐EVs treatment group (Figure S23A–F and Figure 7A,B). Moreover, mitochondrial function was significantly improved and the MAMs formation was inhibited under RA pre‐treatment (Figure 7C). At the same time, the interaction intensity between TDP43 and VDAC1 significantly decreased after RA treatment (Figure S23G,H). In addition, RA administration could also alleviate the severity of atherosclerotic progression under TAR administration, including decreased plaque area/METs formation, increased collagen content, improved inflammatory response, and lipid metabolism disorder (Figure 7D–H and Figure S24). These findings indicated that RA could inhibit METs formation by suppressing TAR‐EVs‐mediated mitochondrial‐ER coupling and calcium overload, thus attenuating atherosclerotic lesions.

## 4 Discussion

Smoking accelerates the progression of AS plaques and increases the instability, thereby promoting plaque rupture and coronary thrombosis [3]. Nevertheless, the precise mechanism through which AS plaques are exacerbated by smoking remains to be fully elucidated [43]. ECs and macrophages are pivotal in the progression of plaques. In the current study, we investigate the mechanisms by which smoking promotes the progression of atherosclerosis, focusing on the interaction of ECs and macrophage communication. The principal findings are as follows: (1) Cigarette TAR facilitates the development of AS via promoting the secretion of EVs by ECs. (2) TAR enhanced METs formation by promoting the secretion of TDP43‐carrying EVs from ECs. (3) TDP‐43 in EVs promotes the formation of MAMs by binding to VDAC1, causing mitochondrial calcium overload, and subsequently facilitating the formation of METs. (4) Targeted inhibition of TDP‐43 in ECs significantly ameliorates the progression of AS plaques induced by cigarette TAR. The current study broadens our understanding of the mechanisms by which smoking promotes the progression of AS, and identifies novel targets for the prevention and treatment of smoking‐associated AS.

In recent years, several studies have elucidated the mechanisms by which smoking contributes to the progression of AS plaques. A significant portion of prior research has concentrated on nicotine, with less research on cigarette TAR. Yang et al. demonstrated that nicotine may facilitate the progression of AS by increasing reactive oxygen species levels and activating the NLRP3 inflammasome [44]. Similarly, Zhang et al. indicated that nicotine induces endothelial cells to mesenchymal transition via the α7nAChR‐ERK1/2‐Snail signaling pathway to promote the progression of AS [45]. Cigarette TAR is also the main toxic component of cigarette smoke. However, in recent years, studies focusing on cigarette TAR and AS plaques have been relatively few. Our research team has reported that cigarette TAR directly acts on smooth muscle cells and macrophages, leading to programmed necrosis, this process enhances the local inflammatory response within the atherosclerotic plaque and promotes plaque progression in ApoE^−/−^ mice [46]. In the current study, we observed that cigarette TAR can stimulate the secretion of EVs by ECs, and EVs inhibitor, GW4869, significantly mitigates the progression of AS plaques induced by cigarette TAR. These results suggest that the intercellular communication mediated by ECs may play a crucial role in the smoking‐related progression of AS plaques. Previous studies have also elucidated the mechanism by which smoking accelerates the progression of AS through EVs. For instance, Tian et al. reported that nicotine facilitates AS progression by increasing EVs carrying miR‐155 and activating the NF‐κB pathway, thereby enhancing ECs inflammation [32]. The current results elucidate the mechanism by which TAR promotes AS progression by augmenting communication between ECs and macrophages. Our results contribute to a deeper understanding of the mechanisms underlying smoking‐related AS progression.

Macrophages are a crucial component of the immune system, playing a pivotal role in immune responses by providing protection against infection and regulating tissue inflammation [47]. Upon exposure to various pathogenic and pro‐inflammatory stimuli, macrophages release extracellular traps content MPO and cell‐free DNA to eliminate pathogens [27]. Furtherer study has confirmed that METs play a significant role in aseptic inflammatory disease [14]. AS is a chronic vascular inflammatory disease, the role and mechanism of METs in AS remain largely unclear. It has been observed that the expression of METs markers was elevated in the plaques of AS patients [48]. Moreover, exogenous inhibition of METs significantly improves ApoE^−/−^ vascular plaque area and lipid deposition [49]. Our study demonstrated that the expression of METs markers, MPO and citH3, was increased in the plaque of ApoE^−/−^ mice in the cigarette TAR treatment group. It was also found that EVs derived from vascular ECs after cigarette TAR treatment significantly increased the expression of METs‐related markers in macrophages in vitro. Conversely, the application of the inhibitor GW4869 significantly diminished the expression of METs markers MPO and citH3 in EVs derived from vascular ECs exposed to cigarette TAR. Consequently, we have substantiated that EVs originating from TAR‐treated ECs trigger the formation of METs. These findings offer novel evidence implicating METs in the progression of smoking‐related AS.

TDP43 is a multifunctional nucleic acid‐binding protein that participates in RNA splicing, transport, and stability, which plays a crucial role, thereby significantly influencing regulating gene expression, especially closely related to neurodegenerative diseases, particularly Alzheimer disease [34]. Previous studies have demonstrated that TDP43 is involved in the regulation the inflammatory response [37, 50]. Nevertheless, its involvement in the inflammatory response associated with AS and the underlying mechanisms remain unclear. Our proteomics data revealed that TDP43 is increased in EVs derived from ECs exposed to cigarette TAR as well as in serum from smoking patient and mice. Silencing TDP43 in ECs can inhibit the formation of METs and significantly markedly decelerate the progression of atherosclerotic plaques caused by cigarette TAR. In the in vitro study, our findings indicated that treatment with cigarette tar did not result in a statistically significant increase in the TDP43 content of EVs derived from hepatocytes, which are the primary source of EVs in circulation, as well as from vascular smooth muscle cells and macrophages, which are cells integral to plaque formation (Figure S25). These results emphasize the pivotal role of TDP43 in mediating the interaction between ECs and macrophages, which triggers the formation of METs. As METs represent a crucial mechanism for modulating the inflammatory response, our study enhances the understanding of how TDP43 regulates the inflammation and immune response. Moreover, in our small‐scale clinical study, we found that TDP43 levels were significantly increased in circulating EVs from smoking patients. Notably, elevated TDP43 levels in circulating EVs were observed to correlate with MACE. These data are preliminary and insufficient to support the conclusion that TDP43 predicts MACE events in STEMI patients. Nevertheless, further studies with larger sample sizes and external validation cohorts are needed to confirm these findings. Our research elucidates the role of TDP43 in the progression of atherosclerotic plaques and suggests that TDP43 may be a potential intervention target for smoking‐induced progression of atherosclerotic plaques.

Endoplasmic reticulum‐mitochondria‐associated membranes (MAMs) represent the functional contact sites between the endoplasmic reticulum and the mitochondria, facilitating inter‐organelle communication [51]. MAMs not only serve as a structural bridge between the mitochondria and the endoplasmic reticulum, but also extensively participate in the regulation of mitochondrial biosynthesis and function, calcium (Ca^2+^) homeostasis, apoptosis, autophagy, lipid metabolism, and oxidative stress responses [52]. In recent years, increasing attention has been directed toward the involvement of MAMs in cardiovascular diseases. Chen et al. have demonstrated the regulatory effects of MAMs on the proliferation of cardiac cells and cardiac regeneration [53]. In the present study, it was observed that TDP43 within EVs binds to VDAC1 and promotes the formation of MAMs in macrophages. The endoplasmic reticulum serves as the main site for calcium ion storage within the cell. Prior research has demonstrated that MAMs are the principal mechanism facilitating the transfer of calcium ions from the endoplasmic reticulum to the mitochondria, leading to calcium overload in the mitochondria [54, 55]. Furthermore, cellular calcium overload or mitochondrial calcium overload can act as an inducer, activating Peptidyl arginine deiminase 4, which in turn leads to the formation of the extracellular traps [56, 57]. Based on these findings, we hypothesize that TDP43 in EVs may enhance the formation of MAMs, thereby causing calcium overload and ultimately facilitating the formation of METs. It was found that EVs could elevate the calcium ion level in macrophages, while the knocking down of TDP43 could significantly inhibit MAMs and calcium ion overload. Furthermore, BAPTA, a calcium ion chelator, and 2‐APB, a specific inhibitor of IP3R, significantly reduced the formation of METs. These evidences indicate that TDP3/VDAC1‐mediated MAMs are a crucial step in the formation of METs.

The current study is subject to several limitations. First, the association between TDP43 levels in circulating EVs and MACE is based solely on a single‐center, small‐sample study, lacking validation from large‐scale studies or external cohorts. This limitation will be addressed in subsequent research endeavors. Second, although we found that RA binds to TDP43 using molecular docking and dynamic simulation approaches, these findings do not directly confirm that TDP43 binds to RA. Future investigations should aim to elucidate the specific mechanisms through which RA interacts with TDP43 and inhibits its function. Third, a significant limitation of the current study pertains to the experimental design employed for scRNA‐seq. Specifically, the scRNA‐seq library was generated using pooled aortic tissues from 15 ApoE^−/−^ mice, aggregated into a single sample for both the HD group and the HD+TAR group, without the inclusion of independent biological replicate samples for sequencing. While pooled scRNA‐seq is appropriate for exploratory classification of cell subtypes and preliminary visualization of cell‐cell interaction landscapes, this approach lacks the statistical robustness necessary to reliably validate differential gene expression and significant pathway activation between groups. Consequently, the findings derived from the scRNA‐seq dataset in this study are interpreted solely as exploratory descriptive observations, rather than as conclusive evidence of TAR‐induced signaling alterations. The core mechanistic conclusions of this study are substantiated by bulk RNA‐seq, multi‐omics proteomics, and independently replicated in vitro cell and in vivo animal functional assays, all of which include sufficient biological replicates to provide robust statistical support for our proposed pathogenic axis. Fourthly, a notable limitation of this study is the reliance on in silico molecular docking and molecular dynamics simulations to suggest a physical interaction between RA and TDP43, although we conducted cellular thermal shift assay, drug affinity responsive target stability, and micro‐scale thermophoresis, the current study is still without corroborating these findings through more direct biochemical experimental validation techniques such as surface plasmon resonance, or pull‐down assays. Although computational modeling provides valuable preliminary insights into the potential binding interface and stable complex conformation, these in silico results do not constitute definitive evidence of a physical interaction. Consequently, the therapeutic implications of RA in the context of smoking‐associated AS, as discussed in this study, should be considered as hypothesis‐generating rather than as established therapeutic conclusions. Future research involving comprehensive biochemical and structural biology experiments is essential to confirm the RA‐TDP43 binding event and to elucidate its causal role in RA‐mediated vascular protection. Fifthly, in this study, we did not conduct the corresponding rescue experiment of TDP43 at the animal level, which may represent a limitation. Future in vivo rescue experiments are necessary to comprehensively elucidate the pivotal role of TDP43 in the formation of METs during the progression of cigarette tar‐mediated AS progression. Sixthly, in the present study, we performed EVs detection on cell supernatants and observed no statistically significant difference in TDP43 levels between the CTRL‐AAV‐Tie2‐shTDP43 and TAR‐AAV‐Tie2‐shTDP43 groups. Furthermore, upon exposure of liver cells, smooth muscle cells, and macrophages to TAR, the TDP43 levels in the EV supernatants remained unchanged statistically. While these findings indirectly suggest that circulating TDP43 primarily originates from ECs, definitive evidence necessitates the use of multiple flow cytometry antibodies to identify the principal source of TDP43 at the in vivo level through flow cytometry. However, our study is limited by the absence of an in vivo flow cytometry platform for detecting EV levels, which represents a constraint of this research and underscores the need for further investigation in future studies.

## 5 Conclusion

Cigarette TAR promotes AS progression by enhancing METs formation and inflammatory response via EVs‐packaged TDP43 derived from ECs, the underlying mechanism is that TDP43 increases MAMs formation and triggers calcium overload by binding to VDAC1 in macrophages. We also discovered that RA may serve as a putative protective molecule to be verified to inhibit MAMs by binding to TDP43, and improve the progression of METs and AS. Our findings identified a novel potential therapeutic target for smoke‐related AS.

## Author Contributions

Conceptualization: H.J., B.Y. S.H., J.L., and X.L. Methodology: X.Z., X. L., Y.C, B.X., and S.Z. Validation: J.L., X.L. (Xing Luo), Y.L., S.Z, X.Z., X.L. (Xiaolin Liu), and Y.C. Investigation: X.L., M.W., C.Z., M.Z. W.M., and Y.W. Resources: Q.S., S.H., M.L. M.L., and X.L. Writing – Original Draft: X.L. Writing – Review & Editing: J.M., Q.G., M.Z., and S.H. Funding Acquisition: X.L., H.J. Supervision: H.J. and B.Y.

## Funding

This work was supported by National Natural Science Foundation of China (82425030, U24A20648, 82370464 and 820720031 to H.J., 82470341 to S.H., 82500554 to X.L), the National Key R&D Program of China (2023YFC3043504 to H.J.), Natural Science Foundation of Heilongjiang Province (JJ2026BS0173 to X.Z.), Heilongjiang Graduate Excellence Funding Program (LJYXL2024‐018), Key Laboratory of Myocardial Ischemia Open Research Project (KF202313), Harbin Medical University Graduate Research and Innovation Fund (YJSCX2024‐38HYD) and Postdoctoral Research Funding of Heilongjiang Province (LBH‐Z24219 to X.L.) the China Postdoctoral Science Foundation (2025M772156 to X.L.) and Postdoctoral Innovation Talent Support Program (BX20250260 to X.L.).

## Ethics Statement

The study received approval from the Institutional Research Ethics Committee of Harbin Medical University (Approval No. YJSDW2024‐047).

## Conflicts of Interest

The authors declare no conflicts of interest.

## Supporting information




**Supporting File**: advs78001‐sup‐0001‐SuppMat.docx.

## Data Availability

The data that support the findings of this study are available on request from the corresponding author. The data are not publicly available due to privacy or ethical restrictions.
