# Supplementary material for: Extracellular Vesicle Packaged TDP43 Derived From ECs Exacerbates Cigarette Tar‐Related Atherosclerosis Progression via Enhancing Macrophage Extracellular Traps
Source: Adv Sci (Weinh). 2026 Sep 27:e78001. Online ahead of print. doi: 10.1002/advs.78001 (PMC13616328; doi:10.1002/advs.78001)
Supplement: Supplementary file 1 [file ADVS-9999-e78001-s001.docx]

**Extracellular vesicle packaged TDP43 derived from ECs exacerbates cigarette tar-related atherosclerosis progression via enhancing macrophage extracellular traps**

Xinxin Zhu ^a, b, 1^, Yuwu Chen ^a, b, 1^, Wei Meng ^a, b^, Biyi Xu ^a, b, 1^, Junke Mou ^a, b, 1^, Xiaolin Liu ^a, b, 1^, Mengyang Wang ^a, b^, Qishuo Gu ^a, b^, Qianhui Sun ^a, b^, Man Li ^a, b^, Chen Zhao ^a, b^, Ming Zeng ^a, b^, Ying Lv ^a, b^, Boling Yi ^a, b^, Sheng Qu ^a, b^, Xiaoxuan Bai ^a, b^, Ji Li ^a, b^ , Xing Luo ^a, b, *^, Sining Hu ^a, b, *^, Bo Yu ^a, b^, Haibo Jia^a, b, *^

^a^ Department of Cardiology of the Second Affiliated Hospital, Harbin Medical University, State Key Laboratory of Frigid Zone Cardiovascular Diseases (SKLFZCD), No. 246 Xuefu Road, Nangang District, Harbin 150001, China.

^b^ The Key Laboratory of Myocardial Ischemia, Chinese Ministry of Education, Harbin, 150001, PR China.

1 These authors contributed equally to the work.

***Address for correspondence:**

Dr. Jia Department of Cardiology, 2nd Affiliated Hospital of Harbin Medical University, Harbin 150001, PR China; National Key Laboratory of Frigid Zone Cardiovascular Diseases (NKLFZCD), Harbin 150001, China

Email: [luoxing321@163.com](mailto:luoxing321@163.com); [hsn_831027@163.com](mailto:hsn_831027@163.com); [haibojia@hrbmu.edu.cn](mailto:haibojia@hrbmu.edu.cn);

**Methods**

**Animals’ study**

**1.Design**

(1) The first section: The ApoE^-/-^ mice were randomly divided into three groups and were given different administrations for a duration of 16 weeks: **①*HD group***: high fat diet (HD) (21% fat, 0.15% cholesterol; MD12015; Medicine Ltd., Jiangsu, China) (HD group), N=6; **②*TAR group***: HD supplemented an intraperitoneal injection of cigarette TAR (40 mg/kg/day) [1-2] , N=6; **③*Tar+GW4869 group***: On the basis of the TAR group, 2 mg/kg of GW4869 was administered intraperitoneally every week for 12 weeks, with 3 injections each week (TAR+GW4869 group) [3] , N=6.

(2) The second section: The ApoE^-/-^ mice were randomly divided into two groups and were given different administrations for a duration of 16 weeks: **①*CTRL ApoE^-/-^ EVs group***: Based on the HD diet administration, the ApoE^-/-^ mice were injected with ECs-derived EVs via the tail vein. In detail, the ECs-derived EVs is the EVs in the ECs supernatant treated with the PBS for 24 hours (The treated volume was the same as the TAR volume used in the TAR-EVS-treated group), N=6; The specific injection dose and frequency are: 20 μg EVs dissolved in 100 μL PBS, once weekly injection. **②*TAR ApoE^-/-^ EVs group***: Based on the HD diet administration, the ApoE^-/-^ mice were injected with ECs-derived EVs via the tail vein. In detail, the ECs-derived EVs is the EVs in the ECs supernatant treated with the 100μg/ml TAR [2], N=6; The specific injection dose and frequency are: 20 μg EVs dissolved in 100 μL PBS, once weekly injection.

(3) The third section: The ApoE^-/-^ mice were randomly divided into four groups and were given different administrations for a duration of 16 weeks: **①*PBS group***: Based on the HD diet administration, the ApoE^-/-^ mice were injected with sterile PBS (same volume as the CTRL EVs group) via the tail vein, N=6; **②*CTRL EVs group***: Based on the HD diet administration, the ApoE^-/-^ mice were injected with ECs-derived EVs via the tail vein. In detail, the ECs-derived EVs is the EVs in the ECs supernatant treated with the PBS for 24 hours(The treated volume was the same as the TAR volume used in the TAR-EVS-treated group), N=6; The specific injection dose and frequency are: 20 μg EVs dissolved in 100 μL PBS, once weekly injection; **③*si-NC-TAR-EVs group:*** Based on the HD diet administration, the ApoE^-/-^ mice were injected with ECs-derived EVs via the tail vein. In detail, the ECs-derived EVs is the EVs in the ECs supernatant treated with the 100μg/ml TAR [2] and si-NC, N=6; The specific injection dose and frequency are: 20 μg EVs dissolved in 100 μL PBS, once weekly injection; **④*si-TDP43-TAR-EVs group:*** Based on the HD diet administration, the ApoE^-/-^ mice were injected with ECs-derived EVs via the tail vein. In detail, the ECs-derived EVs is the EVs in the ECs supernatant treated with the 100μg/ml TAR [2] and si-TDP43, N=6; The specific injection dose and frequency are: 20 μg EVs dissolved in 100 μL PBS, once weekly injection;

(4) The fourth section: The ApoE^-/-^ mice were randomly divided into four groups and were given different administrations for a duration of 16 weeks: **①*****CTRL+AAV-Tie2-sh-NC group***: Based on the HD diet administration, the ApoE^-/-^ mice were injected with adeno-associated virus via the tail vein, in detail, the mice were received intravenous injection of AAV carrying the negative control (NC) plasmids (100 μL vehicle containing 1.0×10^12^ AAV2/9 vector particles, one-time injection); N=6; **②*CTRL+AAV-Tie2-sh-TDP43 group***: Based on the HD diet administration, the ApoE^-/-^ mice were injected with adeno-associated virus via the tail vein, in detail, the mice were received intravenous injection of AAV carrying the sh-TDP43 plasmids (100 μL vehicle containing 1.0×10^12^ AAV2/9 vector particles, one-time injection); N=6; **③*****TAR+AAV-Tie2-sh-NC group***: Based on the HD diet and TAR administration, the ApoE^-/-^ mice were injected with adeno-associated virus via the tail vein, in detail, the mice were received intravenous injection of AAV carrying the negative control (NC) plasmids (100 μL vehicle containing 1.0×10^12^ AAV2/9 vector particles, one-time injection); N=6; **④*TAR+AAV-Tie2-sh-TDP43 group:*** Based on the HD diet and TAR administration, the ApoE^-/-^ mice were injected with adeno-associated virus via the tail vein, in detail, the mice were received intravenous injection of AAV carrying the sh-TDP43 plasmids (100 μL vehicle containing 1.0×10^12^ AAV2/9 vector particles, one-time injection); N=6;

(5) The fifth section: The ApoE^-/-^ mice were randomly divided into two groups and were given different administrations for a duration of 16 weeks: **①*PBS+TAR-EVs group***: Based on the HD diet and TAR-EVs administration, the ApoE^-/-^ mice were received PBS oral administration, N=6; **②*RA+TAR-EVs group***: Based on the HD diet and TAR-EVs administration, the ApoE^-/-^ mice were received RA (5mg/kg/day) [4] oral administration, N=6;

After 16 weeks, the aortas of the ApoE^-/-^ mice were removed after terminal anesthesia (1.5% isoflurane inhalation) and euthanasia by cervical dislocation. The plasma was collected and frozen immediately. The hearts and aortas were removed carefully and fixed with 4% paraformaldehyde (4 °C for 24 h) or kept in optimal cutting temperature compound (OCT) for immunostaining.

**2. H&E, Masson and Oil red O staining**

The ApoE^-/-^ mice were euthanized and after the sample collection, the aortic root tissues were fixed with 4% paraformaldehyde, then embedded in paraffin and cut into 5-micron thick sections. The degree of atherosclerosis and the morphology and size of atherosclerotic plaques were evaluated using Oil Red O staining (Solarbio, China) and Hematoxylin-Eosin (H&E) staining (Solarbio, China) according to the manufacturer’s instructions. The collagen deposition was assessed using Masson’s trichrome staining (Solarbio, China), and the stained sections were analyzed using ImageJ software.

**3. Enzyme-linked immunosorbent assay (ELISA)**

Plasma samples were collected from different groups of ApoE^-/-^ mice for a period of 16 weeks. The concentrations of IL-1β, IL-18, IL-6, TNF-α, IL-4 and IL-10 in the plasma of ApoE^-/-^ mice were determined using an ELISA kit (Nanjing Jiancheng Biotechnology Research Institute, China). The concentrations of the above cytokines were further detected by ELISA analysis (Bost Biotechnology Co., Ltd.) and the levels of cytokines were calculated using ELISA calc software based on the standard curve.

**4. Lipid content evaluation**

Referring to prior published studies [5], fresh mouse blood samples were first kept at room temperature for 2 h. Afterwards, centrifugation was performed at 4 °C and 3000 rpm for 10 min, followed by serum separation. Serum lipid concentrations were determined with commercial detection kits, including ab285242 for total cholesterol (TC), ab65336 for triglyceride (TG), and ab65390 for high-density lipoprotein (HDL) as well as low-density lipoprotein (LDL) measurement.

**5. Immunofluorescence staining**

After processing, the aortic tissues were fixed with 4% PFA for 15 minutes, then rinsed 3 times with PBS solution. A 0.03% Triton X-100 permeabilization treatment was performed. The samples were blocked with BSA (1%) at room temperature for 60 minutes. Then, the samples were incubated with the primary antibody at 4°C overnight. After 3 rinses with PBS solution, the samples were incubated with the corresponding secondary antibodies (1:200, Abcam, USA) at room temperature for 1 hour to visualize the specific proteins. The cell nuclei were counterstained with DAPI for 5 minutes, and the cytoskeleton was counterstained with Phalloidin. Images were obtained using a laser confocal microscope (Leica, Wetzlar, Germany). The detailed antibodies were listed in the ***Supplementary Table 1***.

Image acquisition, standardized imaging parameters, and post-processing were conducted using a Leica SP5 laser scanning confocal microscope (Leica, Wetzlar, Germany) for all fluorescence images. To prevent inter-group signal bias, consistent hardware settings-including laser power, photomultiplier tube gain, exposure time, pinhole size, and Z-step interval-were rigorously maintained across all matched experimental groups for each target protein. For quantitative analysis, a minimum of five random non-overlapping visual fields per tissue section were imaged using the same magnification objective. Raw, unadjusted confocal image files were stored as original source data in a lossless .lif format and preserved for future validation.

Quantitative image analysis, including fluorescence co-localization and relative signal intensity quantification, was conducted using ImageJ software version 1.54f. Prior to quantitative measurement, no global adjustments to brightness or contrast were applied; instead, a linear uniform brightness normalization was implemented across all groups following the extraction of raw signal values. Statistical comparisons were performed using mean fluorescence intensity values derived from unaltered raw pixel data. All original raw imaging files will be made available upon reasonable request.

**6. Western Blot**

Extract total protein from mouse macrophage of aortas using RIPA lysis buffer containing 1 millimole of PMSF (Beiotium, China). After measuring and adjusting the protein concentration using BCA kit (Beiotium, China), the protein samples were separated by SDS polyacrylamide gel electrophoresis. Then, the proteins were transferred onto 0.22 μm PVDF membrane (product of Millipore, USA). After the membrane transfer was completed, the membrane was incubated with 5% skim milk at room temperature for 1 hour continuously shaken. After the incubation, the membrane and antibodies were incubated at 4°C overnight, then incubated with secondary antibody at room temperature for 1 hour continuously shaken. Finally, the membrane was stained with ECL kit (Solectron, China). Add anti-TDP43 antibody (1:50) or normal rabbit IgG (1:50) as a control to the supernatant, and incubate these two mixtures overnight on a roTARy shaker at 4°C. Add 20 μl of protein A+G magnetic beads (product of Thermo Fisher, USA) to each mixture and incubate for 2 hours together at 4°C. Perform agarose bead precipitation and 3 washing steps at 4°C to complete antibody capture. Elute the proteins by heating at 95°C for 5 minutes. Finally, analyze the immunoprecipitated proteins by SDS-PAGE and immunoblotting. The detailed antibodies were listed in the ***Supplementary Table 2***.

7. **Isolation of Macrophages from Arterial Tissue**

Macrophages were isolated from freshly obtained arterial tissues. Briefly, the tissues were sectioned and subjected to collagenase digestion. F4/80 microbeads (MACS Miltenyi Biotec, Catalog No. 130-110-443, San Diego, CA) were utilized to isolate macrophages from the tissue homogenates. Initially, F4/80-positive cells were magnetically labeled with F4/80 microbeads. The cell suspension was subsequently applied to a MACS Column positioned within the magnetic field of a MACS separator. The magnetically labeled F4/80-positive cells were retained within the column. Upon removal of the column from the magnetic field, the magnetically retained F4/80-positive cells were eluted as the positively selected cell fraction. The F4/80 microbead-labeled cells were then cultured in RPMI 1640 medium supplemented with 10% fetal bovine serum (FBS) and subsequently used for expression analysis.

**8. EVs extraction**

In view of the previous literature [6], Mouse blood was collected via intracardiac puncture into 1.5 ml Eppendorf tubes containing trisodium citrate as an anticoagulant. Plasma was isolated by centrifuging whole blood at 1,600×g for 15 min at room temperature. Purified plasma was either immediately centrifuged for EVs isolation or stored at -80 °C. Plasma EVs were obtained by ultracentrifugation: briefly, plasma was diluted 1:1 with PBS and subjected to sequential centrifugation at 4 °C: 500×g for 5 min, 3,000×g for 25 min, 12,000×g for 60 min, and finally 120,000×g for 70 min (polycarbonate tubes, 355630; MLA-55 rotor, Optima MAX-XP, Beckman Coulter). The EV pellet was collected, and the remaining supernatant was processed using the Total Exosome Isolation Kit (from plasma; Invitrogen, 4484450) per the manufacturer’s guidelines. Briefly, 0.2 volumes of Exosome Precipitation Reagent were added to the supernatant, incubated at room temperature for 10 min, then centrifuged at 10,000×g for 30 min at 4 °C to separate EVs from the supernatant. The resulting EV pellets were collected and resuspended in PBS.

**9. qPCR**

Total ribonucleic acid (RNA) was isolated from treated murine macrophage of aorta using Trizol reagent (Invitrogen, USA). Complementary DNA (cDNA) was synthesized with a Reverse Transcription Kit (Roche, Switzerland), followed by reverse transcription-polymerase chain reaction (RT-PCR) using Synergetic Binding Reagent Green (Roche, Switzerland) to quantify the mRNA expressions of TDP43 and MPO. The specific primers used are listed in the ***Supplementary Table 3***, and all data were normalized to the mRNA levels of glyceraldehyde-3-phosphate dehydrogenase (GAPDH), which served as the endogenous reference gene.

The detailed experimental procedures are described as follows:

(1) For tissue samples, 200 mg of tissue was weighed and directly mixed with 1 mL of Trizol; the tissue was thoroughly homogenized using a high-throughput tissue oscillator at 50 Hz, with three cycles of 1 minute each (total 3 minutes). Subsequent RNA extraction steps were consistent for both cell and tissue samples: 1) Add 200 μL of chloroform and vortex vigorously to mix thoroughly. 2) Centrifuge at 1789×g for 15 minutes at 4°C. 3) Carefully transfer the upper colorless aqueous phase into a new Eppendorf (EP) tube. 4) Add an equal volume of isopropanol, mix gently by inverting the tube, and centrifuge at 1789×g for 15 minutes at 4°C. Discard the supernatant, and the precipitate at the bottom of the tube is the RNA. 5) Add 75% ethanol to wash the RNA precipitate, pipette gently to resuspend the precipitate, centrifuge at 1789×g for 5 minutes at 4°C, and discard the supernatant. 6) Add 100% ethanol for a second wash, centrifuge at 1789×g for 5 minutes at 4°C, and discard the supernatant. 7) Add an appropriate volume of diethylpyrocarbonate (DEPC)-treated water to dissolve the RNA, and then determine the RNA concentration using a UV spectrophotometer.

(2) After RNA extraction, residual genomic DNA (gDNA) was eliminated by treatment with DNase I (1 U/μg RNA) at 37°C for 30 minutes, followed by heat inactivation to terminate the reaction. The absence of gDNA contamination was verified by including no-RT controls in the RT-PCR assay.

(3) Reverse transcription was performed in accordance with the instructions provided with the Roche Reverse Transcription Kit, using a 10 μL reaction system containing 0.5 μg of total RNA. The reverse transcription reaction was carried out on a PCR instrument with the following program: 25°C for 10 minutes, 50°C for 30 minutes, 85°C for 5 minutes, and then held at 4°C.

(4) For RT-PCR amplification, a 10 μL reaction system was used, consisting of 5 μL of Roche Synergetic Binding Reagent Green, 3.5 μL of DEPC-treated water, 0.25 μL each of the forward and reverse primers, and 1 μL of cDNA template. The amplification program was as follows: initial denaturation at 95°C for 10 minutes, followed by 40 cycles of denaturation at 95°C for 15 seconds and annealing at the corresponding temperature for 30 seconds. A melting curve analysis was performed by heating from 65°C to 95°C. Cycle threshold (Ct) values were collected, and the relative mRNA expression levels were calculated.

In RT-PCR analysis, the comparative Ct (ΔΔCt) method is widely used for quantifying relative gene expression. Briefly, the Ct value of the target gene in each sample is normalized to that of the reference gene (GAPDH) to generate the ΔCt value. Next, the ΔCt value of the experimental sample is compared with that of a calibrator sample (e.g., the control group) to obtain the ΔΔCt value. The relative expression level is then calculated as 2^^(-ΔΔCt)^. This approach compensates for variations in RNA input and reverse transcription efficiency, thereby enabling a normalized and comparative evaluation of gene expression levels. All results were presented as bar graphs.

**10. RNA sequencing (RNA-seq)**

For sample preparation, HD-fed ApoE^-/-^ mice were subjected to HD or HD+TAR exposure over a 16-week period. The aortas were harvested (4 HD mice aorta vs 4 HD+TAR aorta), immediately frozen in liquid nitrogen, and preserved at -80 °C until further use. Total RNA was extracted from the frozen aortic tissues using the RNeasy Mini kit (QIAGEN, catalog no. 74104) following the manufacturer’s instructions. Prior to library construction, RNA quality control and quantification were conducted: RNA concentration was measured with the Qubit RNA kit (Life Technologies), and RNA integrity was evaluated using the Agilent Bioanalyzer. Only samples with a RNA Integrity Number (RIN) greater than 7 were selected for subsequent experiments. cDNA libraries were constructed from 100 ng of total RNA using the Stranded Total RNA prep with Ribo-Zero Plus kit (Illumina), accompanied by 13 cycles of PCR amplification. The final cDNA libraries were subjected to size validation via either the Agilent Bioanalyzer or Tapestation, and their concentrations were determined by quantitative PCR (qPCR) using reagents from Kapa Biosystems/Roche. Libraries were then normalized, pooled together, denatured with 0.2 N NaOH, and diluted to a final concentration of 250 pM. The pooled libraries were loaded onto an Illumina NovaSeq V1.5 cartridge for cluster generation, followed by sequencing on an Illumina NovaSeq 6000 platform using a paired-end 101-bp sequencing strategy, aiming to obtain approximately 30-40 million reads per sample. For bioinformatics analysis, raw RNA-seq reads were first subjected to quality filtering, and then aligned to the mouse reference genome (mm10) using the STAR aligner (version 2.7.9a). Uniquely mapped reads were quantified with HTSeq (version 0.11.0), and only protein-coding genes with non-zero read counts were included for downstream analyses. Differential gene expression analysis was performed using the DESeq2 software (version 1.36.0); genes with an adjusted *P*-value < 0.05 and a fold change (FC) > 2 were defined as significantly differentially expressed genes (DEGs). Unsupervised clustering was employed to generate heat maps using the heatmap. Gene Ontology (GO) enrichment analysis of DEGs was carried out using the enrich GO tool within the cluster profiler package (version 4.4.4).

**11. Single cell RNA sequencing (scRNA-seq)**

**①The preparation of aortas**: As reported in prior studies[7], after ApoE^-/-^ mice were euthanized, whole aortas (including the adventitia) were carefully dissected to strip off adipose tissue and collected. The isolated aortas were immediately placed on ice in tissue preservation solution (Miltenyi Biotec), with 15 blood vessels per centrifuge tube. Subsequently, the aortas were sectioned and digested at 37°C in Hank’s Balanced Salt Solution supplemented with 450 U/mL Collagenase I (Sigma Aldrich), 250 U/mL Collagenase XI (Sigma Aldrich), 120 U/mL Hyaluronidase (Sigma Aldrich), and 120 U/mL DNase I (Worthington), with reciprocal shaking at 60 rpm for 1 hour. The resulting cell suspension was filtered through a 40 μm cell strainer (BD Biosciences), and red blood cells were eliminated using Red Cell Lysis Buffer. The remaining cells were rinsed with pre-warmed Dulbecco’s Modified Eagle Medium (DMEM) containing 10% fetal bovine serum at 37°C, centrifuged at 500×g for 5 minutes, and resuspended in phosphate-buffered saline for subsequent scRNA-seq analysis.

**②Quality control and data normalization:** Following the manufacturer’s instructions, 30 aortas from ApoE^-/-^ mice (15 fed HD and 15 fed an HD diet plus TAR administration) were processed using the 10×Genomics Chromium Single Cell 3’ Reagent Kits v2. Aortas were pooled in groups of 15 to form individual samples, resulting in a total of 2 samples (1 per group: HD and HD+TAR) for subsequent single-cell RNA sequencing (scRNA-seq) analysis. Cell Ranger v9.0.1 was used to process barcodes and quantify single-cell 3’ gene counts[8], with further analyses performed using the Seurat R package. Low-quality cells were first filtered out based on the following criteria: nFeature_RNA ≥ 200, nCount_RNA ≥ 1000, percent.mito < 20%, and log10GenesPerUMI ≥ 0.7. Doublet cells were removed using DoubletFinder, and data normalization was conducted to standardize sequencing depth across all cells.

**③Dimension reduction**: Dimensionality reduction was conducted in three sequential steps: identification of 2000 variable genes, Principal Component Analysis (PCA), and Uniform Manifold Approximation and Projection (UMAP)[9]. Variable genes were identified using the FindVariableGenes function in the Seurat R package. The fastMNN algorithm was used to mitigate batch effects during PCA[10]. Next, bidimensional coordinates for each cell were generated via the RunUMAP function. The FindNeighbors function was applied to determine k-nearest neighbors for each cell, enabling construction of the Shared Nearest Neighbor (SNN) graph. Finally, cells were clustered into distinct groups using the FindClusters function with a resolution parameter of 0.4.

**④Cellchat analysis**: Using CellChat (v1.4.0) [11], we inferred cell-cell interactions among ECs, fibroblasts, smooth muscle cells (SMCs), T/NK cells, Macrophages, Neutrophils, B cells. Following the CellChat tutorial, we conducted a comparative analysis between the CTRL and TAR groups. We assessed communication probabilities to identify up- and down-regulated ligand-receptor signaling pairs in each group.

**12. The evaluation of serum cell free DNA**

Blood samples were collected into EDTA-containing tubes and processed within 4 hours. Plasma was isolated via two rounds of centrifugation at 2000 g for 10 minutes at 4°C, aliquoted into 1 mL tubes, and stored at -80°C until further analysis. Circulating cell-free DNA (cfDNA) concentrations were quantified using the Qubit Fluorometric Quantitation double-stranded (ds) DNA Assay Kit (Thermo Fisher Scientific).

**Cell studies**

1. **Cell culture**

The human monocytic THP-1 cells (provided by the ATCC institution in the United States) were cultured in RPMI 1640 medium (provided by Gibco in the United States), with 10% fetal bovine serum (provided by Gibco in the United States) and 1% penicillin (provided by Beo-Taim in China) added to the medium, and cultured in an environment at 37°C with 5% carbon dioxide saturation. HUVECs (purchased from the National Certified Cell Culture Repository in Shanghai, China) were cultured in the Dubecco modified Eagle's medium (DMEM; Gibco in New York State, Grand Isle Island) with 10% fetal bovine serum (FBS; Scientific Cell Company, San Diego) in an environment at 37°C with 5% carbon dioxide saturation. 100 micrograms/ml of cigarette TAR was used to stimulate human umbilical vein endothelial cells (HUVECs) for 24 hours.

**2. JC-1 staining**

JC-1 staining working solution was prepared according to the instructions. The JC-1 staining working solution was added and the culture plate was placed in a 37°C incubator for incubation for 15-20 minutes. After incubation, the cells were washed twice with JC-1 buffer (1×) and observed under a fluorescence microscope. When the cell function was impaired, the JC-1 staining of mitochondrial membrane potential would show a decrease in red fluorescence and an increase in green fluorescence, indicating the loss of mitochondrial membrane potential and possible mitochondrial dysfunction.

**3. Measurement of intracellular and mitochondrial Ca^2+^**

The intracellular and mitochondrial calcium ion concentrations were measured using Fluo-4, AM (Beiyue Biology, China) and Rhod-2, AM (Maokang Biology, China), respectively. The staining was performed according to the operation manual. After staining, the cells were washed slowly with PBS solution three times, then incubated with the working solution in a 37°C incubator for 30 minutes. After the incubation, the cells were washed again with PBS solution three times. The observation was conducted using a laser scanning confocal microscope.

**4. Detection of ROS**

The measurement of total reactive oxygen species (ROS) was carried out using DCFH-DA (Bay Biosciences, China) through fluorescence microscopy. The cells were seeded in 24-well plates, washed twice with PBS solution, and then incubated with the loading buffer containing freshly prepared probes (10 μM) for 30 minutes. The fluorescence intensity was measured using a fluorescence microscope.

**5. Western blot and immunoprecipitation**

Extract total protein from treated cells using RIPA lysis buffer containing 1 millimole of PMSF (Beiotium, China). After measuring and adjusting the protein concentration using BCA kit (Beiotium, China), the protein samples were separated by SDS polyacrylamide gel electrophoresis. Then, the proteins were transferred onto 0.22 μm PVDF membrane (product of Millipore, USA). After the membrane transfer was completed, the membrane was incubated with 5% skim milk at room temperature for 1 hour continuously shaken. After the incubation, the membrane and antibodies were incubated at 4°C overnight, then incubated with secondary antibody at room temperature for 1 hour continuously shaken. Finally, the membrane was stained with ECL kit (Solectron, China). Add anti-TDP43 antibody (1:50) or normal rabbit IgG (1:50) as a control to the supernatant, and incubate these two mixtures overnight on a roTARy shaker at 4°C. Add 20 μl of protein A+G magnetic beads (product of Thermo Fisher, USA) to each mixture and incubate for 2 hours together at 4°C. Perform agarose bead precipitation and 3 washing steps at 4°C to complete antibody capture. Elute the proteins by heating at 95°C for 5 minutes. Finally, analyze the immunoprecipitated proteins by SDS-PAGE and immunoblotting. The detailed description of the antibodies is listed in the ***Supplementary Table 2***.

**6. Immunofluorescence staining**

After processing, the cells were fixed with 4% PFA for 15 minutes, then rinsed 3 times with PBS solution. A 0.03% Triton X-100 permeabilization treatment was performed. The samples were blocked with BSA (1%) at room temperature for 60 minutes. Then, the samples were incubated with the primary antibody at 4°C overnight. After 3 rinses with PBS solution, the samples were incubated with the corresponding secondary antibodies at room temperature for 1 hour to visualize the specific proteins. The cell nuclei were counterstained with DAPI for 5 minutes, and the cytoskeleton was counterstained with Phalloidin. Images were obtained using a laser confocal microscope (Leica, Wetzlar, Germany). The detailed description of the antibodies is listed in the ***Supplementary Table 1***.

Cellular fluorescence images were acquired using a Leica SP5 laser scanning confocal microscope (Leica, Wetzlar, Germany). Consistent instrumental parameters, including laser wavelength and power, photomultiplier tube (PMT) gain, exposure time, pinhole size, objective magnification, and Z-stack interval, were meticulously maintained across all staining batches to prevent artificial signal bias. To ensure unbiased quantification, a minimum of five random, non-overlapping visual fields were captured per biological replicate well. The unaltered raw imaging files were saved in a lossless .lif format and comprehensively archived as original source data, which are available upon reasonable request.

A quantitative analysis of relative fluorescence intensity and co-localization signals was conducted utilizing ImageJ software, version 1.54f. Prior to the quantification of pixel intensity, no non-linear modifications, such as arbitrary brightness or contrast enhancement, were applied to the raw images. Instead, a uniform linear normalization was implemented following the extraction of raw signal values to facilitate group comparisons. Co-localization coefficients between target proteins were determined using threshold-masked raw fluorescence signals.

**7. EVs extraction and co-culture with THP-1 cells**

We isolated and purified EVs from HUVECs using the differential centrifugation method. The cells were cultured in serum-free conditions and stimulated with or without TAR and 100 micrograms/ml of cigarette TAR for 48 hours. The supernatant was collected and centrifuged at 3000×g for 15 minutes at 4°C to separate the samples and remove cells and debris. Then, 1 milliliter of the sample was mixed with 10 milliliters of PBS solution for dilution. The sample was centrifuged at 10,000×g for 15 minutes at 4°C. The supernatant was transferred to an ultracentrifuge tube and centrifuged at 100,000×g for 1 hour at 4°C to obtain a white or clear precipitate. To further purify, the precipitate was washed with PBS and then centrifuged at 100,000×g for 1 hour at 4°C to obtain purified EVs for subsequent studies. Protein markers, such as Alix, TSG101, CD63, and calnexin were detected by western blotting. 25 μg/mL EVs were cultured with THP-1 for performing functional assays.

**8. Knock down of TDP43 expression by shRNA**

To knockdown TDP43 expression, shRNA targeting TDP43 was used. Scramble shRNA (sh-NC) and TDP43 shRNA were obtained from Invitrogen (Carlsbad, CA, USA). Macrophages were seeded in 6‑well plates and transfected with 50 nM sh-NC or sh-TDP43 using Lipofectamine 3000 reagent (Invitrogen).

**9. qPCR**

Total ribonucleic acid (RNA) was isolated from treated THP-1 using Trizol reagent (Invitrogen, USA). Complementary DNA (cDNA) was synthesized with a Reverse Transcription Kit (Roche, Switzerland), followed by reverse transcription-polymerase chain reaction (RT-PCR) using Synergetic Binding Reagent Green (Roche, Switzerland) to quantify the mRNA expressions of TDP43 and MPO. The specific primers used are listed in the ***Supplementary Table 3***, and all data were normalized to the mRNA levels of glyceraldehyde-3-phosphate dehydrogenase (GAPDH), which served as the endogenous reference gene.

The detailed experimental procedures are described as follows:

(1) For cell samples, 200 mg of cell was weighed and directly mixed with 1 mL of Trizol; Subsequent RNA extraction steps were consistent for both cell and tissue samples: 1) Add 200 μL of chloroform and vortex vigorously to mix thoroughly. 2) Centrifuge at 1789×g for 15 minutes at 4°C. 3) Carefully transfer the upper colorless aqueous phase into a new Eppendorf (EP) tube. 4) Add an equal volume of isopropanol, mix gently by inverting the tube, and centrifuge at 1789×g for 15 minutes at 4°C. Discard the supernatant, and the precipitate at the bottom of the tube is the RNA. 5) Add 75% ethanol to wash the RNA precipitate, pipette gently to resuspend the precipitate, centrifuge at 1789×g for 5 minutes at 4°C, and discard the supernatant. 6) Add 100% ethanol for a second wash, centrifuge at 1789×g for 5 minutes at 4°C, and discard the supernatant. 7) Add an appropriate volume of diethylpyrocarbonate (DEPC)-treated water to dissolve the RNA, and then determine the RNA concentration using a UV spectrophotometer.

(2) After RNA extraction, residual genomic DNA (gDNA) was eliminated by treatment with DNase I (1 U/μg RNA) at 37°C for 30 minutes, followed by heat inactivation to terminate the reaction. The absence of gDNA contamination was verified by including no-RT controls in the RT-PCR assay.

(3) Reverse transcription was performed in accordance with the instructions provided with the Roche Reverse Transcription Kit, using a 10 μL reaction system containing 0.5 μg of total RNA. The reverse transcription reaction was carried out on a PCR instrument with the following program: 25°C for 10 minutes, 50°C for 30 minutes, 85°C for 5 minutes, and then held at 4°C.

(4) For RT-PCR amplification, a 10 μL reaction system was used, consisting of 5 μL of Roche Synergetic Binding Reagent Green, 3.5 μL of DEPC-treated water, 0.25 μL each of the forward and reverse primers, and 1 μL of cDNA template. The amplification program was as follows: initial denaturation at 95°C for 10 minutes, followed by 40 cycles of denaturation at 95°C for 15 seconds and annealing at the corresponding temperature for 30 seconds. A melting curve analysis was performed by heating from 65°C to 95°C. Cycle threshold (Ct) values were collected, and the relative mRNA expression levels were calculated.

In RT-PCR analysis, the comparative Ct (ΔΔCt) method is widely used for quantifying relative gene expression. Briefly, the Ct value of the target gene in each sample is normalized to that of the reference gene (GAPDH) to generate the ΔCt value. Next, the ΔCt value of the experimental sample is compared with that of a calibrator sample (e.g., the control group) to obtain the ΔΔCt value. The relative expression level is then calculated as 2^^(-ΔΔCt)^. This approach compensates for variations in RNA input and reverse transcription efficiency, thereby enabling a normalized and comparative evaluation of gene expression levels. All results were presented as bar graphs.

**10. LC-MS analysis**

LC-MS data processing was performed using ultra-high-performance liquid chromatography (OE Biotech Co., Ltd.). Briefly, stable THP-1 expressing TDP43-Flag were lysed with NP-40 lysis buffer (Beyotime). Whole-cell lysates were incubated with 5 µL of anti-Flag antibody (Cell Signaling Technology) or control rabbit IgG at 4°C for 4 hours. After incubation, the mixtures were incubated with 10 µL of Protein A+G agarose beads (Santa Cruz Biotechnology) at 4°C for an additional 1 hour. The immunoprecipitated beads were then analyzed using a Fusion Lumos mass spectrometer (Thermo Fisher Scientific).

**11. Transmission Electron Microscopy (TEM)**

EVs were fixed in 2% paraformaldehyde, adsorbed onto 200-mesh Formvar‑coated copper grids, negatively stained with 2% phosphotungstic acid, and examined under a transmission electron microscope (TEM; H-7650, Hitachi, Tokyo, Japan).

**12. NTA**

EVs were exposed to laser irradiation to record their Brownian motion. A 10‑second video was captured and analyzed using nanoparticle tracking analysis (NTA) software (version 2.3, NanoSight, Salisbury, UK). In addition, a Nano Analyzer system (NanoFCM, model N30E) was employed to perform nanoflow cytometry for particle characterization.

**13. MAMs staining analysis by Immunofluorescence**

THP-1 were seeded into confocal chambers and stained with Mito‑Tracker (1:1000 dilution) and ER-Tracker (1:1000 dilution) at 37 °C for 30 minutes. Mitochondrial morphology was observed and imaged under a confocal laser scanning microscope (Leica SP5, Wetzlar, Germany). Mitochondrial length was quantified using ImageJ software equipped with the Mitochondrial Network Analysis (MiNA) toolset.

**14. SYTOX Green staining**

To precisely quantify the formation of METs, SYTOX Green was employed for live cell imaging. Cells cultured in 24-well plates underwent various treatments, followed by incubation with 1 µM SYTOX Green for 20 minutes. Subsequently, the cells were examined using a fluorescence microscope.

15. **Protease protection assay**

In the protease protection assays, EVs were partitioned into four groups. EVs were subjected to treatment with 1% Triton X-100 (EMD-Millipore) and 1 μg/ml trypsin (Promega). The Triton X-100 treatment was conducted on ice for a duration of 30 minutes, followed by trypsin treatment at 25°C for 1 hour. All samples were incubated under identical temperature conditions and durations.

**16.Over expression of TDP43**

To achieve TDP43 overexpression, a eukaryotic expression plasmid carrying full-length TDP43 coding sequence was utilized. TDP43 overexpression plasmid (OE-TDP43) were purchased from Invitrogen (Carlsbad, CA, USA). ECs were seeded in 6-well plates and transfected with equal amounts of OE-TDP43 using Lipofectamine 3000 reagent (Invitrogen).

**17.Molecular docking analysis**

The molecular structures of TDP43 and VDAC1 were retrieved from the PubChem database (https://pubchem.ncbi.nlm.nih.gov/). The energy minimization of TPD43 was conducted utilizing the MMFF2 force field in conjunction with the Powell conjugate gradient minimization algorithm, applying a convergence criterion of 0.005 kcal/(mol•Å). Subsequently, Discovery Studio (DS) 2.5 was employed to delineate the active site of the NRF2 crystal structure and to compute the docking site, which was encapsulated within a grid box measuring 18.6 Å×18.6Å×18.6Å. TDP43 was designated as the ligand for the VDAC1 protein, and molecular docking was executed at varying binding ratios using the LibDock method. The docking energies were subsequently calculated to assess the binding affinity of TDP43 to VDAC1.

18. **Construction of Wild-Type VDAC1 and Truncated VDAC1-Δ Expression Vectors**

Full-length VDAC1-WT (aa 1-283) was PCR-amplified from human cDNA and cloned into Flag-tagged expression vector. The truncated VDAC1-Δ mutant lacking residues 146-210 was constructed by amplifying two separate fragments covering aa 1-145 and aa 211-283, followed by ligation into the same Flag vector. All constructs, together with HA-tagged full-length TDP43 plasmid, were sequence-verified prior to endotoxin-free plasmid preparation for cell transfection.

19. **Cellular Thermal Shift Assay (CETSA)**

THP-1 cells were incubated with or without RA at a concentration of 100 μM for a duration of 24 hours. Following incubation, the cells were harvested and subjected to the CETSA as previously described [12]. Briefly, the incubated cells were partitioned into 11 equal aliquots, each of which was exposed to a 3-minute heat treatment at varying temperatures: 37, 41, 45, 49, 53, 57, 61, 65°C. Post-heating, the cells were frozen at -80 °C for 12 hours, followed by a 5-minute incubation at room temperature; this freeze-thaw cycle was repeated once. Subsequently, cell lysates were prepared by centrifugation at 20,000 × g for 20 minutes, and the expression levels of TDP43 were evaluated through WB analysis.

**20.Drug affinity responsive target stability (DARTS)**

In the DARTS protocol, extracts obtained from THP-1 cells were incubated with or without RA at a concentration of 100 μM, following previously established procedures [12]. The incubation was conducted for 24 hours at room temperature. Following incubation, the lysates were partitioned into six separate aliquots, each subjected to digestion with varying concentrations of pronase E (Sigma-Aldrich). The digestion process was carried out for 20 minutes at room temperature. To terminate the digestion, a loading buffer was added, and the samples were immediately boiled. Subsequently, the treated samples were individually loaded onto a 10% SDS-PAGE gel for WB analysis.

**Clinical studies**

**1. Clinical Sample**

(1) The plasma EVs were isolated using an ultra-high-speed centrifugation method and were used for detected TDP43 level (n=9), proteomics analysis (n=10) or treated macrophages(n=10). All patients signed the informed consent form.

(2) STEMI was defined as continuous chest pain for > 30 min, ST-segment elevation > 0.1 mV in at least two contiguous leads, or new left bundle-branch block on the 12-lead electrocardiogram (ECG), and elevated cardiac markers (troponin T/I or creatine kinase-MB). 5ml peripheral blood were collected from these patients prior to discharge for the isolation of circulating EVs. TDP43 expression in peripheral blood EVs was detected using ELISA. The major adverse cardiac events (MACE) was a composite of all-cause death, recurrent nonfatal MI, nonfatal stroke, or hospitalization due to heart fail. In this cohort, after matching for age, gender, hypertension, diabetes, and hyperlipidemia, there were 30 smokers and 30 non-smokers, and the levels of TDP43 in the circulating EVs were compared. All patients signed the informed consent form.

(3) A total of 20 patients who visited the Neurosurgery Department of the Second Affiliated Hospital of Harbin Medical University and were diagnosed with carotid artery stenosis were included from 2024 to 2025. These patients underwent carotid endarterectomy, and the resected carotid plaques were collected. They were divided into two groups based on smoking status (smokers, n=10 and nonsmokers, n=10), with matching for risk factors including age, gender, hypertension, hyperlipidemia, and diabetes. All patients signed the informed consent form.

**Clinical/Animal/Cell studies**

**1. EVs Proteomics**

EVs were isolated from three independent levels: Plasma EVs from 10 smoking vs. 10 non‑smoking CHD patients; Plasma EVs from ApoE^-/-^ mice with vs. without TAR exposure (n=6 per group); EVs from HUVECs treated with or without TAR (100 μg/mL, 24 h) (n=3 biological replicates); The specific analysis process is listed as follows:

**①Sample preparation for proteomic analysis**: In view of the previous literature [13], all EVs samples were resuspended in lysis buffer (PBS pH 7.4) supplemented with 7 M Urea, 2 M Thiourea, and 1×complete protease inhibitor, then sonicated at 4°C for 3 min (5 s pulse, 10 s interval, 50% power). After centrifugation at 14,000×g for 20 min at 4°C, the lysate supernatant was collected. Proteins were reduced with 10 mM DTT at 37°C for 1 h, then alkylated with 20 mM IAA in the dark under the same conditions. Samples were buffer-exchanged to 50 mM ABC using Amicon Microcon centrifugal filters (10 kDa cutoff) before being digested overnight at 37°C with trypsin (1:50 enzyme-to-protein mass ratio) and lyophilized for subsequent use.

**②Quality assessment of proteomic data:** All data analyses in this section were performed in R, including assessment of batch effects and quantitative stability of sample proteomics data. Proteome datasets were filtered to retain proteins with more than two unique peptides, then normalized using the normalize Between Arrays function from the limma R package (v3.6.3), with missing values imputed as zero. For differential abundance analysis, proteins with >50% missing values across all samples were excluded (only those with ≥50% valid values were included). Protein intensities were log2-transformed prior to subsequent statistical and bioinformatics analyses.

**③Determination of differentially abundant proteins:** Differentially abundant proteins were identified via the Wilcoxon rank-sum test using the rstatix R package (v0.7.2). Statistical significance for up- and down-regulation was defined as p < 0.05, with median log_2_ fold changes (log_2_FC) of ±0.58 further classifying these significant changes.

**④Quantification:** For protein quantification, we performed label‑free quantitative proteomic analysis on EVs derived from three independent sources: smoking versus non‑smoking patients, TAR‑exposed versus control ApoE^-/-^ mice, and TAR‑treated versus untreated ECs. Protein abundances were determined based on the intensities of unique peptides, and only proteins detected with at least two unique peptides were included for reliable quantification. Relative protein levels were calculated as normalized spectral counts or intensity‑based absolute quantification to ensure comparability across all samples and groups.

**⑤Normalization:** Raw proteomic data were subjected to strict normalization procedures to minimize technical variation. Protein intensity values were log2‑transformed to approximate a normal distribution, and between‑array normalization was applied using the limma package in R to adjust for global differences in sample loading and instrumental response. Missing values were imputed using the k‑nearest neighbor method, and low‑confidence proteins with more than 50% missing values were excluded to maintain data robustness.

**⑥Statistical thresholds:** Differentially abundant proteins were defined using robust and consistent statistical thresholds across all three cohorts. Proteins with a Wilcoxon rank‑sum test P-value < 0.05 and a |log2(fold change)| ≥ 0.58 (corresponding to a linear fold change ≥ 1.5 or ≤ 0.67) were considered significantly regulated. These thresholds were uniformly applied to patient, mouse, and cellular EV proteomes to ensure fair and reproducible identification of overlapping and treatment‑associated proteins.

**⑦Validation strategy:** A multi‑step validation strategy was implemented to confirm the proteomic findings. The key overlapping protein TDP43 was validated at the protein level by Western blotting in EVs from patients, mice, and endothelial cells, Functional validation was further performed using TDP43 knockdown *in vitro* and *in vivo*, which reversed the pro‑atherogenic and MET‑promoting effects of TAR‑mediated EVs, confirming the specificity and biological relevance of the proteomic discovery.

2. **EV proteomics analytical pipeline**

**①Data normalization and missing value filtering:** All peptide intensity values underwent log_2_ transformation, and inter-array normalization was conducted using the limma package in R. Proteins exhibiting more than 50% missing intensity values across all samples were excluded from subsequent analyses to ensure reliable quantification.

**②Statistical test and FDR multiple testing correction:** The differential abundance between paired groups was assessed using two-sided Wilcoxon rank-sum tests. To control the false discovery rate (FDR), raw p-values were adjusted using the Benjamini–Hochberg procedure. Proteins exhibiting an FDR-adjusted p-value of less than 0.05 and an absolute log_2_(fold change) of at least 0.58 (corresponding to a linear fold change of ≥1.5 or ≤0.67) were classified as significantly differentially abundant proteins.

**③Overlap screening criteria across three independent EV cohorts:** Three distinct EV proteomic datasets were independently generated: (1) circulating EVs from coronary artery disease (CAD) patients who smoke compared to non-smoking counterparts; (2) plasma EVs from apolipoprotein E-deficient (ApoE^-/-^) mice exposed to TAR versus control mice; and (3) EVs secreted by human umbilical vein endothelial cells (HUVECs) treated with TAR compared to untreated controls. Proteins identified as significant differentially abundant proteins (DAPs) across all three datasets were retained as core candidate mediators.

**④TDP43 statistical validation after FDR correction:** TDP43 satisfied both FDR-adjusted p < 0.05 and |log_2_FC| ≥ 0.58 thresholds in all three independent EV proteomic cohorts after Benjamini-Hochberg multiple testing correction, confirming its consistent upregulation across patient, animal and cellular levels.

**⑤Venn diagram plotting:** The Venn plot **(Supplementary Figure 8)** was generated based on the above uniform FDR and fold-change cut-offs to visualize shared significant DAPs across the three cohorts.

3. **Sample Size and Experimental Replicates**

All experiments were performed with independent biological replicates, defined as experiments conducted on separate batches of cells, individual animals, or distinct human patient samples. Technical replicates (repeated measurements of the same biological sample) were used to assess intra-assay variability, but only biological replicates were used for statistical analysis:

**①*In vivo* animal experiments (histological staining, serum cytokine analysis):** n=6 mice per group;

**②Western blot analysis:** n=3-5 independent biological replicates per group;

**③Immunofluorescence staining:** n=3-6 independent biological replicates per group, with at least 5 random fields quantified per sample

**④Cellular functional assays (calcium imaging, ROS detection, mitochondrial membrane potential):** n=5 independent biological replicates per group

**⑤Clinical sample analysis:** n=10 patients per group for proteomics and histological analysis; n=30 patients per group for ELISA and survival analysis

**Statistical analysis**

Statistical analyses were performed with GraphPad Prism 9 (GraphPad Software, San Diego, CA, USA). All the results were presented as mean ± standard error of mean, and all the data were acquired from at least three independent experiments. Two-tailed Student’s t-test were used to compare data between two groups with normal distributed values. Mann–Whitney U test was used to compare data between two groups with non-normal distributed values. For survival analysis, time-to-first event curves were generated by Kaplan-Meier analysis, and compared using the log-rank test. In addition, univariate and multivariable Cox proportional-hazards regression models were used to estimate hazard ratios (HRs) and 95% confidence intervals (CI). Logistic regression model was used to analysis the association between EVs TDP43 level and MACE. Statistical analysis was performed using SPSS 21.0 software. P < 0.05 was considered statistically significant.

**Supplemental Fig. 1. TAR stimulation improved lipid levels in ApoE^-/-^ mice.**

(A) Serum total cholesterol levels (N=5); (B) Serum triglyceride levels (N=5); (C) Serum low-density lipoprotein levels (N=5); (D) Serum high-density lipoprotein levels (N=5); Results are expressed as mean±SD. Comparisons of parameters were performed with one-way ANOVA with Bonferroni post hoc test. ^****^*P*＜0.0001;

**Supplemental Fig 2. TAR stimulate the secretion of EVs by ECs**

(A) Endothelial cells were divided in two groups and stimulated by PBS and TAR respectively. Ultrastructure of CTRL-EVs and TAR-EVs were observed through TEM; (B) Western blot analysis revealed that the expression of Alix, TSG101, CD63, Calnexin and ApoA1 (N=3); (C) Size distribution and particle concentration of EVs;

**Supplemental Fig. 3. Cigarette TAR stimulation promotes the expression of extracellular traps formation markers.** (A). PCA distribution of the RNA-seq analysis; (B). Volcano plot of differentially expressed genes between the control group and the cigarette tar-stimulated group; (C). The log_2_Foldchange and q-value of the differential expressed genes; (D) GO enrichment analysis of up-regulated genes between the control group and the cigarette TAR-stimulated group; (E) HE staining of carotid plaques from smoking and non-smoking patients; (F) Immunofluorescence analysis of MPO levels in macrophages from smoking and nonsmoking patients; Results are expressed as mean±SD. Comparisons of parameters were performed with Student’s t-test. ^*^*P*＜0.05, ^****^*P*＜0.0001.

；

**Supplemental Fig. 4. Co-staining of phalloidin and fluorescently labeled EVs via** **phalloidin immunofluorescence staining.**

**Supplemental Fig. 5. Lipid content evaluation in CTRL ApoE^KO^ EVs group and TAR ApoE^KO^ EVs group.** (A) Serum total cholesterol levels (N=6); (B) Serum triglyceride levels (N=6); (C) Serum low-density lipoprotein levels (N=6); (D) Serum high-density lipoprotein levels (N=6); Results are expressed as mean±SD. Comparisons of parameters were performed with Student’s t-test.

**Supplemental Fig. 6. The quality control process of the scRNA-seq data.**

(A) Sequencing parameters of 10× Genomics scRNA-seq platform; (B) Distribution of gene numbers (nFeature_RNA), UMI (nCount_RNA) and percentage of mitochondrial genes (percent.mt) per cell from raw data; n=1 biological sample per group (15 aortas pooled).

**Supplemental Fig. 7. The umap visualization and cellchat analysis of the scRNA-seq data.**

(A) UMAP plots of different cell types in CTRL and TAR group; (B) The proportion of the different cell types in CTRL and TAR group; (C) The dot plot of the different cell types in CTRL and TAR group; (D) Circle plots showing interaction strength in CTRL and TAR group; Only one pooled biological sample (15 aortas pooled) was sequenced per group, without independent replicates; cell-cell interaction circle plots are for exploratory description only.

**Supplemental Fig. 8. TDP43 is a key protein mediating the production of EVs from endothelial cells stimulated by cigarette TAR.**

**Supplemental Fig. 9. TDP43 is a key protein that mediates the production of EVs by endothelial cells stimulated by TAR in the animal, cellular, and patient levels.** (A-B) Expression level of TDP43 in plasma EVs from Smoker versus Nonsmoker assessed by Western blot (N=10); (C, F and I) Total RNA was extracted from the cells to detect MPO and CitH3 mRNA levels (N=10: Patients/animal; N=5: Cells;); (D-E) Expression of TDP43 in EVs from the serum of control and cigarette TAR stimulated mice assessed by Western blot (N=6); (G-H) Expression of TDP43 in EVs from the supernatant of control and cigarette TAR-stimulated endothelial cells assessed by Western blot (N=5); Results are expressed as mean±SD. Comparisons of parameters were performed with Student’s t-test. ^***^*P* < 0.001, ^****^*P* < 0.0001.

**Supplemental Fig. 10. The protease protection assays under TX-100 and Trypsin treatment.**

**Supplemental Fig. 11. The staining of SYTEX green under sh-TDP43 administration (N=5).** Results are expressed as mean±SD. Comparisons of parameters were performed with one-way ANOVA with Bonferroni post hoc test. ^****^*P* < 0.0001.

**Supplemental Fig. 12. TDP43 carried by EC-derived EVs promotes METs formation.** (A). EVs derived from cigarette TAR-stimulated ECs were isolated and incubated with macrophages; (B). Total RNA was extracted from the cells to detect MPO mRNA levels (N=6); (C). MPO serum level evaluation via ELISA (N=6); (D-E). Western blot analysis and quantification of the protein expression levels of MPO, CitH3, and TDP43 (N=5); (F-I). Co-immunofluorescence staining of MPO/CitH3 and DAPI (N=6); (J-K). The staining of SYTOX green (N=6); (L-M). Co-localization staining of ER-tracker and Mito-tracker (N=6); Results are expressed as mean±SD. Comparisons of parameters were performed with one-way ANOVA with Bonferroni post hoc test. ^****^*P* < 0.0001.

**Supplemental Fig. 13. Lipid content evaluation in PBS/CTRL-EVs/si-NC-TAR-EVs/si-TDP43-TAR-EVs group.** (A) Serum total cholesterol levels (N=6); (B) Serum triglyceride levels (N=6); (C) Serum low-density lipoprotein levels (N=6); (D) Serum high-density lipoprotein levels (N=6); Results are expressed as mean±SD. Comparisons of parameters were performed with one-way ANOVA with Bonferroni post hoc test.

**Supplemental Fig. 14. Levels of cell-free DNA in the plasma of ApoE^-/-^ mice.** N=5. Results are expressed as mean±SD. Comparisons of parameters were performed with one-way ANOVA with Bonferroni post hoc test. ^****^*P* < 0.0001.

**Supplemental Fig. 15.** (A-B) Western blot detection and quantification of Alix and TDP43 protein expression (N=5). Results are expressed as mean±SD. Comparisons of parameters were performed with Student’s t-test. ^****^*P* < 0.0001.

**Supplemental Fig. 16. mRNA content detection of TDP43 in different cell subsets via flow cytometry.** (A) The gating strategy of ECs via flow cytometry; (B). TDP43 mRNA content of ECs in AAV-tie2-sh-NC and AAV-tie2-sh-TDP43 group, (N=10); (C) The gating strategy of macrophages via flow cytometry; (D). TDP43 mRNA content of macrophages in AAV-tie2-sh-NC and AAV-tie2-sh-TDP43 group, (N=10); (E) The gating strategy of SMCs via flow cytometry; (F). TDP43 mRNA content of SMCs in AAV-tie2-sh-NC and AAV-tie2-sh-TDP43 group, (N=10); Results are expressed as mean±SD. Comparisons of parameters were performed with Student’s t-test. ^****^*P* < 0.0001.

**Supplemental Fig. 17. sh-TDP43 abolishes the atherosclerotic effects of TAR in ApoE^-/-^ mice. ApoE^-/-^ mice were randomly assigned to four groups and intraperitoneal injection of PBS and cigarette TAR. Each group received either AAV-sh-NC or AAV-sh-TDP43 via tail vein injection. After 16 weeks, arterial tissues were harvested for analysis.** (A-B) Representative images of H&E and Masson staining of aortic root sections, with quantitative analysis of plaque size and collagen area (N = 6); (C) Oil Red O staining and quantification of lipid deposition on the aortic surface (N = 6); (D) Serum levels of IL-1β, IL-6, TNF-α, IL-18, IL-6, IL-4 and IL-10 were measured by ELISA (N = 6); (E-F) Immunofluorescence results show co-localization of MPO, CitH3 and CD68 (N=3); (G) ELISA detection of TDP43 levels in circulating EVs (N=10). NTA analysis of the number of EVs in circulation (N=10); (H-I) Western blot detection and quantification of MPO and CitH3 protein expression (N=3); (J) Levels of cell-free DNA in the plasma of ApoE^-/-^ mice (N=5); Results are expressed as mean±SD. Comparisons of parameters were performed with one-way ANOVA with Bonferroni post hoc test. ^*^*P* < 0.05, ^**^*P* < 0.01, ^****^*P* < 0.0001.

**Supplemental Fig. 18. Lipid content evaluation in CTRL-AAV-tie2-sh-NC/CTRL-AAV-tie2-sh-TDP43/TAR-AAV-tie2-sh-NC/TAR-AAV-tie2-sh-TDP43 group.** (A) Serum total cholesterol levels (N=6); (B) Serum triglyceride levels (N=6); (C) Serum low-density lipoprotein levels (N=6); (D) Serum high-density lipoprotein levels (N=6); Results are expressed as mean±SD. Comparisons of parameters were performed with one-way ANOVA with Bonferroni post hoc test. ^****^*P*＜0.0001;

**Supplemental Fig. 19. TDP43 knockdown could inhibit the formation of METs.**

(A) Co-immunoprecipitation was used to evaluate the binding of TDP43 and VDAC1 (N=3); (B-E) Western blot detection and quantification of MPO and CitH3 expression (N=5); (F). Co-localization staining of ER-tracker and Mito-tracker (N=6); (G-H).The staining of SYTEX green; Results are expressed as mean±SD. Comparisons of parameters were performed with one-way ANOVA with Bonferroni post hoc test. ^****^*P* < 0.0001.

**Supplemental Fig. 20. The oxidative stress level evaluation under VBIT4/TDP43 knockdown/BAPTA/2-APB administration.** (A). The MDA content under VBIT-4 and si-TDP43 treatment (N=5); (B). The GSH content under VBIT-4 and si-TDP43 treatment (N=5); (C). The SOD activity under VBIT-4 and si-TDP43 treatment (N=5); (D). The 4-HNE content under VBIT-4 and si-TDP43 treatment (N=5); (E). The MDA content under BAPTA and 2-APB treatment (N=5); (F). The GSH content under BAPTA and 2-APB treatment (N=5); (G). The SOD activity under BAPTA and 2-APB treatment (N=5); (H). The 4-HNE content under BAPTA and 2-APB treatment (N=5); Results are expressed as mean±SD. Comparisons of parameters were performed with one-way ANOVA with Bonferroni post hoc test. ^**^*P* < 0.01, ^***^*P* < 0.001, ^****^*P* < 0.0001.

**Supplemental Fig. 21. The 145-211 site of VDAC1 is linked to TDP43 and mediates the formation of MAMs.** (A-B). The molecular docking analysis of VDAC1 and TDP43 interaction; (C). Schematic representation of VDAC1 truncates; (D-E). The Co-IP analysis of VDAC1 and TDP43 interaction; (F-G). Co-localization staining of ER-tracker and Mito-tracker (N=6); Results are expressed as mean±SD. Comparisons of parameters were performed with one-way ANOVA with Bonferroni post hoc test. ^****^*P* < 0.0001.

**Supplemental Fig. 22. RA directly interacts with TDP43;** (A) Lysates from THP-1 cells were incubated with or without RA (100 µM) for 24 h. Different concentrations of pronase E were added for 20 min, and TDP43 content was analyzed using WB analysis; (B) Lysates from THP-1 cells were incubated with RA at the indicated concentrations for 24 h, with a final concentration of 0.01% pronase E added for 20 min. The level of TDP43 was assessed through WB analysis. (C) THP-1 cells were incubated with RA (100 µM) for 24 h. These samples were then analyzed using CETSA. Values were expressed as mean ± SD (n = 4). (E) MST demonstrating a direct interaction between RA and EGFP-tagged TDP43 in lysates from EGFP-TDP43 expressing HEK293T cells. Results are expressed as mean±SD. Comparisons of parameters were performed with Student’s t-test (A, C). Comparisons of parameters were performed with one-way ANOVA with Bonferroni post hoc test (B). ^*^*P* < 0.05, ^***^*P* < 0.001, ^****^*P* < 0.0001.

**Supplemental Fig. 23. RA administration could inhibit the formation of METs.** (A-B). Western blot detection and quantification of MPO and CitH3 expression (N=6); (C-D) Co-localization staining of ER-tracker and Mito-tracker (N=6); (E-F). The staining of SYTEX green (N=6); (G) Co-immunoprecipitation was used to assess the binding of TDP43 to VDAC1 (N=6); Results are expressed as mean±SD. Comparisons of parameters were performed with one-way ANOVA with Bonferroni post hoc test. ^****^*P* < 0.0001.

**Supplemental Fig. 24. Lipid content evaluation in PBS+TAR-EVs/RA+TAR-EVs**

**group.** (A) Serum total cholesterol levels (N=6); (B) Serum triglyceride levels (N=6); (C) Serum low-density lipoprotein levels (N=6); (D) Serum high-density lipoprotein levels (N=6); Results are expressed as mean±SD. Comparisons of parameters were performed with Student’s t-test. ^**^*P*＜0.01; ^***^*P*＜0.001; ^****^*P*＜0.0001;

**Supplemental Fig. 25. TDP43 in serum EVs of different cell types with or without TAR treatment;** (A) TDP43 content in serum EVs of hepatocyte (N=8); (B) TDP43 content in serum EVs of macrophages (N=8); (C) TDP43 content in serum EVs of VSMCs (N=8); Results are expressed as mean±SD. Comparisons of parameters were performed with Student’s t-test.

| **Antibodies** | **Manufacturer** | **Cat.** | **Dilution** |
| --- | --- | --- | --- |
| Anti-Myeloperoxidase antibody[EPR20257] | Abcam | ab208670 | *In vivo*: 1:100  *In vitro*: 1:400 |
| Anti-Histone H3 (citrulline R2 + R8 + R17) antibody [RM1001] | Abcam | ab281584 | *In vivo*: 1:100  *In vitro*: 1:400 |
| Anti-CD68 antibody [RM1031] | Abcam | ab303565 | *In vivo*: 1:100 |

**Supplemental Table 1. The antibodies of Immunofluorescence staining**

| **Antibodies** | **Manufacturer** | **Cat.** | **Dilution** |
| --- | --- | --- | --- |
| Myeloperoxidase Antibody | CST | #79623 | 1:1000 |
| Citrullinated Histone H3 (Arg17) (E4O3F) Rabbit Monoclonal Antibody | CST | #97272 | 1:1000 |
| beta-Actin Antibody | CST | #4967 | 1:1000 |
| TDP43 (D9R3L) Rabbit Monoclonal Antibody | CST | #89789 | 1:1000 |
| GAPDH (14C10) Rabbit Monoclonal Antibody | CST | #2118 | 1:1000 |
| VDAC (D73D12) Rabbit Monoclonal Antibody | CST | #4661 | 1:1000 |
| Alix (3A9) Mouse Monoclonal Antibody | CST | #2171 | 1:1000 |
| TSG101 (E6V1X) Rabbit Monoclonal Antibody | CST | #72312 | 1:1000 |
| CD63 (E1W3T) Rabbit Monoclonal Antibody | CST | #52090 | 1:1000 |
| Calnexin (C5C9) Rabbit Monoclonal Antibody | CST | #2679 | 1:1000 |
| ApoA1 (5F4) Mouse Monoclonal Antibody | CST | #3350 | 1:1000 |
| HRP-conjugated Rabbit anti-Goat IgG (H+L) | Abclonal | AS029 | 1:10000 |
| Goat Anti-Mouse IgG H&L (HRP) | Abcam | Ab97040 | 1:10000 |

**Supplemental Table 2. The antibodies of Western Blot**

| **Primers** | **F(5’-3’)** | **R(5’-3’)** |
| --- | --- | --- |
| MPO (Mouse) | AGGGCCGCTGATTATCTACAT | CTCACGTCCTGATAGGCACA |
| GAPDH (Mouse) | AGGTCGGTGTGAACGGATTTG | GGGGTCGTTGATGGCAACA |
| TDP43 (Mouse) | TCCAGAGGCCGAAACAGAG | CAGCTCCTATTGTTGATAAGCCC |
| TDP43 (Human) | AAGTCAGACGAGAGGGGGTTA | GCCAACTCAACATTGGGCT |
| GAPDH (Human) | GGAGCGAGATCCCTCCAAAAT | GGCTGTTGTCATACTTCTCATGG |

**Supplemental Table 3. The primer used in the present study.**

**Reference**

[1]	X. Bao, X. Luo, X. Bai, Y. Lv, X. Weng, S. Zhang, Y. Leng, J. Huang, X. Dai, Y. Wang, J. Li, H. Jia, Cigarette tar mediates macrophage ferroptosis in atherosclerosis through the hepcidin/FPN/SLC7A11 signaling pathway, *Free radical biology & medicine* **2023**, *201*, 76, <https://doi.org/10.1016/j.freeradbiomed.2023.03.006>.

[2]	X. Bai, Y. Wang, X. Luo, X. Bao, X. Weng, Y. Chen, S. Zhang, Y. Lv, X. Dai, M. Zeng, D. Yang, S. Hu, J. Li, Y. Ji, H. Jia, B. Yu, Cigarette tar accelerates atherosclerosis progression via RIPK3-dependent necroptosis mediated by endoplasmic reticulum stress in vascular smooth muscle cells, *Cell communication and signaling : CCS* **2024**, *22* (1), 41, <https://doi.org/10.1186/s12964-024-01480-6>.

[3]	T. Lallemand, M. Rouahi, A. Swiader, M. H. Grazide, N. Geoffre, P. Alayrac, E. Recazens, A. Coste, R. Salvayre, A. Nègre-Salvayre, N. Augé, nSMase2 (Type 2-Neutral Sphingomyelinase) Deficiency or Inhibition by GW4869 Reduces Inflammation and Atherosclerosis in Apoe(-/-) Mice, *Arteriosclerosis, thrombosis, and vascular biology* **2018**, *38* (7), 1479, <https://doi.org/10.1161/atvbaha.118.311208>.

[4]	M. Kalisz, M. Chmielowska, L. Martyńska, A. Domańska, W. Bik, A. Litwiniuk, All-trans-retinoic acid ameliorates atherosclerosis, promotes perivascular adipose tissue browning, and increases adiponectin production in Apo-E mice, *Scientific reports* **2021**, *11* (1), 4451, <https://doi.org/10.1038/s41598-021-83939-x>.

[5]	M. Yang, J. Zheng, X. Zong, X. Yang, Y. Zhang, C. Man, Y. Jiang, Preventive Effect and Molecular Mechanism of Lactobacillus rhamnosus JL1 on Food-Borne Obesity in Mice, *Nutrients* **2021**, *13* (11), <https://doi.org/10.3390/nu13113989>.

[6]	X. Chen, Y. Luo, Q. Zhu, J. Zhang, H. Huang, Y. Kan, D. Li, M. Xu, S. Liu, J. Li, J. Pan, L. Zhang, Y. Guo, B. Wang, G. Qi, Z. Zhou, C. Y. Zhang, L. Fang, Y. Wang, X. Chen, Small extracellular vesicles from young plasma reverse age-related functional declines by improving mitochondrial energy metabolism, *Nature aging* **2024**, *4* (6), 814, <https://doi.org/10.1038/s43587-024-00612-4>.

[7]	X. Luo, J. Li, Y. Chen, X. Weng, X. Bao, X. Bai, Y. Lv, S. Zhang, X. Zhu, B. Xu, C. Zhao, M. Zeng, T. Wu, Q. Sun, S. Wang, M. Liu, T. Johnson, S. J. White, P. Libby, S. Hu, B. Yu, H. Jia, Cigarette Tar Enhanced ECs Pyroptosis via CAMKII/Drp1/mtDNA: Novel Insight Into the Mechanism of Plaque Erosion, *JACC. Basic to translational science* **2025**, *10* (8), 101283, <https://doi.org/10.1016/j.jacbts.2025.03.015>.

[8]	R. Satija, J. A. Farrell, D. Gennert, A. F. Schier, A. Regev, Spatial reconstruction of single-cell gene expression data, *Nature biotechnology* **2015**, *33* (5), 495, <https://doi.org/10.1038/nbt.3192>.

[9]	D. Kotatha, Y. Torii, K. Shinomiya, M. Ogino, S. Uchida, M. Ishikawa, T. Furuike, H. Tamura, Preparation of thin-film electrolyte from chitosan-containing ionic liquid for application to electric double-layer capacitors, *International journal of biological macromolecules* **2019**, *124*, 1274, <https://doi.org/10.1016/j.ijbiomac.2018.12.006>.

[10]	L. Haghverdi, A. T. L. Lun, M. D. Morgan, J. C. Marioni, Batch effects in single-cell RNA-sequencing data are corrected by matching mutual nearest neighbors, *Nature biotechnology* **2018**, *36* (5), 421, <https://doi.org/10.1038/nbt.4091>.

[11]	S. Jin, C. F. Guerrero-Juarez, L. Zhang, I. Chang, R. Ramos, C. H. Kuan, P. Myung, M. V. Plikus, Q. Nie, Inference and analysis of cell-cell communication using CellChat, *Nature communications* **2021**, *12* (1), 1088, <https://doi.org/10.1038/s41467-021-21246-9>.

[12]	R. Jafari, H. Almqvist, H. Axelsson, M. Ignatushchenko, T. Lundbäck, P. Nordlund, D. Martinez Molina, The cellular thermal shift assay for evaluating drug target interactions in cells, *Nature protocols* **2014**, *9* (9), 2100, <https://doi.org/10.1038/nprot.2014.138>.

[13]	J. Wang, C. Z. Gu, P. X. Wang, J. R. Xian, H. Wang, A. Q. Shang, Y. C. Zhong, W. J. Zheng, J. W. Cheng, W. J. Yang, J. Zhou, J. Fan, W. Guo, X. R. Yang, H. J. Lu, Integrative proteomic profiling of tumor and plasma extracellular vesicles identifies a diagnostic biomarker panel for colorectal cancer, *Cell reports. Medicine* **2025**, *6* (5), 102090, <https://doi.org/10.1016/j.xcrm.2025.102090>.
